# Effects of User Experience in Automated Information Processing on Perceived Usefulness of Digital Contact-Tracing Apps: Cross-Sectional Survey Study

**DOI:** 10.2196/53940

**Published:** 2024-06-25

**Authors:** Tim Schrills, Lilian Kojan, Marthe Gruner, André Calero Valdez, Thomas Franke

**Affiliations:** 1 Institute for Multimedia and Interactive Systems Universität zu Lübeck Lübeck Germany

**Keywords:** COVID-19, contact tracing, user experience, trust, health information processing

## Abstract

**Background:**

In pandemic situations, digital contact tracing (DCT) can be an effective way to assess one’s risk of infection and inform others in case of infection. DCT apps can support the information gathering and analysis processes of users aiming to trace contacts. However, users’ use intention and use of DCT information may depend on the perceived benefits of contact tracing. While existing research has examined acceptance in DCT, automation-related user experience factors have been overlooked.

**Objective:**

We pursued three goals: (1) to analyze how automation-related user experience (ie, perceived trustworthiness, traceability, and usefulness) relates to user behavior toward a DCT app, (2) to contextualize these effects with health behavior factors (ie, threat appraisal and moral obligation), and (3) to collect qualitative data on user demands for improved DCT communication.

**Methods:**

Survey data were collected from 317 users of a nationwide-distributed DCT app during the COVID-19 pandemic after it had been in app stores for >1 year using a web-based convenience sample. We assessed automation-related user experience. In addition, we assessed threat appraisal and moral obligation regarding DCT use to estimate a partial least squares structural equation model predicting use intention. To provide practical steps to improve the user experience, we surveyed users’ needs for improved communication of information via the app and analyzed their responses using thematic analysis.

**Results:**

Data validity and perceived usefulness showed a significant correlation of *r*=0.38 (*P*<.001), goal congruity and perceived usefulness correlated at *r*=0.47 (*P*<.001), and result diagnosticity and perceived usefulness had a strong correlation of *r*=0.56 (*P*<.001). In addition, a correlation of *r*=0.35 (*P*<.001) was observed between Subjective Information Processing Awareness and perceived usefulness, suggesting that automation-related changes might influence the perceived utility of DCT. Finally, a moderate positive correlation of *r*=0.47 (*P*<.001) was found between perceived usefulness and use intention, highlighting the connection between user experience variables and use intention. Partial least squares structural equation modeling explained 55.6% of the variance in use intention, with the strongest direct predictor being perceived trustworthiness (β=.54; *P*<.001) followed by moral obligation (β=.22; *P*<.001). Based on the qualitative data, users mainly demanded more detailed information about contacts (eg, place and time of contact). They also wanted to share information (eg, whether they wore a mask) to improve the accuracy and diagnosticity of risk calculation.

**Conclusions:**

The perceived result diagnosticity of DCT apps is crucial for perceived trustworthiness and use intention. By designing for high diagnosticity for the user, DCT apps could improve their support in the action regulation of users, resulting in higher perceived trustworthiness and use in pandemic situations. In general, automation-related user experience has greater importance for use intention than general health behavior or experience.

## Introduction

### Background

During pandemic situations, efficiently acquiring, storing, and evaluating information on physical contacts can be crucial for both individuals and public health agencies aiming to curb infection dynamics [1]. Manual tracing of such contacts is practically impossible, leading to a growing development and research of digital tools supporting such efforts, commonly referred to as digital contact tracing (DCT) apps [2]. By allowing for automation, DCT tools effectively allow for contact tracing. They aim to allow individual users to assess their own risk status with minimal effort and offer support in daily action regulation, such as in decision situations, regarding isolation or notification of previous contacts [3]. If used correctly, DCT can aid in breaking chains of infection and thereby support curbing pandemic spread. For example, in Germany, a DCT called *Corona-Warn-App* (CWA) [4] was developed on behalf of the Federal Ministry of Health, and it was downloaded >40 million times [5].

However, the extent to which individuals use DCT can vary vastly [6]. Previous research has shown that it is crucial whether users perceive a DCT app as beneficial to guide them in pandemic contexts [7]. This core factor is in line with existing models of health behavior (eg, the influential Health Belief Model [HBM] [8]). Within the HBM, perceived benefit is outlined as a central determinant for the implementation of health behavior [7]. When investigating health-related technology, the HBM is frequently connected with models of technology acceptance [9]. As part of these models, the perceived usefulness or performance of technology is similarly postulated as a central variable for use intention. In this paper, we refer to the term *usefulness* as it is better suited than *benefits* to describe the effects of a specific technology. Thereby, we refer to usefulness as “the degree to which a person believes that using a particular system would enhance their [...] performance” [10].

Examining psychological processes revolving around the perception of DCT usefulness is a crucial research topic to understand the adoption and efficient implementation of DCT. Extensive research has shown the importance of the perceived usefulness of DCT for different applications and in different countries [11-15]. All in all, extending existing theoretical approaches such as the HBM by focusing on user experience variables in DCT allows for clear guidelines on improving DCT design and uptake.

The usefulness that a user can experience from DCT results from the automation it provides. DCT takes over tasks that would otherwise need to be done manually (eg, recording contacts, estimating distance and exposure to contacts, and calculating risk based on the vaccination status of contacts). Therefore, it can be defined as an automated system. In general, automation can be defined as a system’s ability to “offload, assist, or replace human performance at corresponding stages of human information processing” [16]. The human action that DCT seeks to automate is the continuous recording and analysis of contact data to monitor an individual’s risk of infection. While there is a large body of research on automation, its adverse biases, and its impact on human performance [17-19], less research focuses on the psychological processes involved when users evaluate the usefulness of automated contact tracing.

Parasuraman et al [20] define 4 evaluation criteria on how automation can affect human performance: situation awareness [21], trust (cf complacency and trust [22]), skill degradation [23], and workload [24]. When users want to make situation-adequate decisions, they benefit from improved situation awareness. Situation awareness, in turn, can be improved by DCT. As long as the information or recommendations provided by DCT apps are perceived as trustworthy, users may use them to determine the right course of action. Accordingly, a DCT’s ability to support situation awareness as well as trust formation (refer to the study by Hoff and Bashir [25]) may lead to perceived usefulness. On the other hand, in the context of DCT apps, one cannot assume that users are potentially losing a previously existing skill through automation; DCT app users are not able to stop sick individuals or themselves. Along the same line, DCT app users profit from automation as it reduces manual work in contact tracing. Therefore, we propose to examine users’ experience of situation awareness and trustworthiness when using DCT apps.

While research has demonstrated that usefulness strongly impacts use intention [26], factors unrelated to the specific DCT app might affect whether people intend to use the system. The HBM positions threat appraisal as another factor directly influencing use intention [7]. While using a DCT app changes neither the susceptibility nor the severity (in comparison, refer to the study by Costa [27]) related to an infection, it is still plausible that users with higher threat appraisal are more interested in their own risk status and, therefore, more likely to use a DCT app (eg, to be able to detect and react to an infection as early as possible). Therefore, threat appraisal may influence use intention independent of the specified design of DCT apps. In addition, recent research has also shown that the theoretical framework of the HBM does profit from incorporating prosocial aspects of decisions [28,29] (ie, using a DCT app may provide a sense of moral obligation to others). Even though individuals with immunity may perceive a lower personal threat, they may feel a personal obligation to track and inform contacts. Overall, to fully investigate the influence of the perceived usefulness of a DCT system on the use intention, a comparison with system-nonspecific factors (ie, threat appraisal) and personal moral obligation should be made. To the best of our knowledge, no previous study has focused on examining the perception of automation-related usefulness while addressing threat appraisal and moral obligation as system-independent factors influencing use intention.

### Research Objective

The objective of this research was to examine how automation-related user experience affects the perceived usefulness of contact tracing as well as use intention of DCT apps and how user experience could be improved. To do so, our approach consisted of multiple methods. The first was quantitatively assessing and analyzing the impact of automation-related user experience (ie, experienced system traceability and perceived trustworthiness) as well as system knowledge on the intention of using a DCT app. The second was contextualizing the effects of automation-related user experience measures with factors related to health protection behavior (ie, threat appraisal and moral obligation). The third was a qualitative analysis of user demands for improved information communication between users and the DCT app. Therefore, the key contribution of this research is a better understanding of how system characteristics lead to perceived usefulness of DCT and how optimal DCT apps can increase use intention through automation-related user experience. Thus, this research supports the human-centered design of DCT apps.

To address these research objectives, 317 users of the CWA DCT system were surveyed about their experience with the app through a web-based questionnaire. A partial least squares structural equation model (PLS-SEM) was used to quantitatively describe the relationships among psychological factors regarding DCT use. This approach was supplemented by a thematic analysis of qualitative user requests on desired communication of information between users and the system.

### Related Research

#### Use Intention of DCT

DCT describes software applications that support documenting information of physical contact or proximity between people (cf [30]). This includes both the (partially) automated acquisition of contact information and the analysis of this information (eg, to determine an individual’s risk of infection [31]). In pandemic situations, users might have the goal to avoid contributing to the further spread of the pandemic disease and, thus, face a control task. This means that users need to constantly self-regulate their actions in relation to their environment (eg, how many people around them are infected). While users strive to achieve this goal, they are constantly facing a changing environment (ie, exposure to infected persons). To maintain control, they need to constantly acquire and analyze information and decide, for example, whether they want to isolate themselves. Such actions taken by users have a profound impact on the trajectory of their individual situation—they potentially curtail further contacts and, thereby, change the future information acquisition process. In this process, DCT constitutes a crucial tool for behavioral control as the information provided functions both as feedback for previous behavior and as an indicator for future behavior.

Although DCT applications, especially on mobile devices, first generated high interest during the COVID-19 pandemic [32], they had already been used previously (refer to, eg, the study by Sacks et al [33]). Due to their wide applicability and potential role in public health systems during the COVID-19 pandemic, research on user behavior toward DCT has increased. Here, diverging acceptance models (such as the Unified Theory of Acceptance and Use of Technology and the technology acceptance model) have been evaluated to understand DCT use intention (eg, the study by Velicia-Martin et al [34]).

As indicated at the outset, previous research on DCT app use has leveraged not only acceptance models but also more general models of health behavior such as the HBM or the Theory of Planned Behavior [35]. Such models have been successfully used in research on the uptake and maintenance of other pandemic protective behaviors. In that context, there is consistent evidence of the importance of factors related to the behavior itself, such as perceived usefulness; factors related to perceived risk, such as threat appraisal; and social and normative factors [11,36]. However, in the DCT context, results are mixed. While there is broad support for the importance of factors such as use intention [35] and perceived usefulness [7], evidence of the role of the other factors is less consistent. For example, Tomczyk et al [35] found evidence of the role of both subjective norms and threat appraisal. In contrast, Walrave et al [7] did not include normative factors in their study and found no significant relationship between threat appraisal and DCT adoption. In a different approach to conceptualizing norms, Zabel et al [37] found a strong association between DCT adoption and moral intensity, a construct that derives the perceived obligation for DCT adoption from a range of beliefs, including beliefs about both usefulness and risk. This not only mirrors findings on the association between moral obligation and other pandemic protective behaviors, but as the community benefit of DCT might outweigh the individual benefit, it also appears to be a promising avenue for exploring the relationship between norms and DCT use. Accordingly, it remains an important task of DCT research to understand the relative influence and interplay of both factors such as perceived usefulness, and factors such as threat appraisal or moral obligation on use intention.

One reason for the ambiguity of existing results can be the variability of operationalizations—trust, for example, is highlighted in multiple studies as decisive for DCT use intention [7,35,37]. However, the conceptualization of trust can be challenging and context-dependent [38]. In DCT, for example, trust could influence one’s belief regarding how effectively DCT can support the individual in avoiding an infection. On the other hand, trust can be related to the data security of private information (refer to, eg, the study by Altmann et al [39]). Therefore, a context-sensitive and theory-based conceptualization of trust is necessary to operationalize it adequately.

#### Breaking Down Automation-Related User Experience in DCT

In a pandemic context, the goal of users can be characterized as behavior that avoids both becoming infected and spreading infection to others. Still, they may desire to meet other people or use public transport and, therefore, are continuously adapting their behavior based on how they perceive the risk situation (ie, for simplification, a perceived risk level; refer to the study by Wilde [40]). This risk level refers to the probability of being infected by, for example, a virus. Acquisition of information on the current risk level is supported by DCT and becomes critical information for comparison, prompting actions to reduce risk.

**Figure 1:**
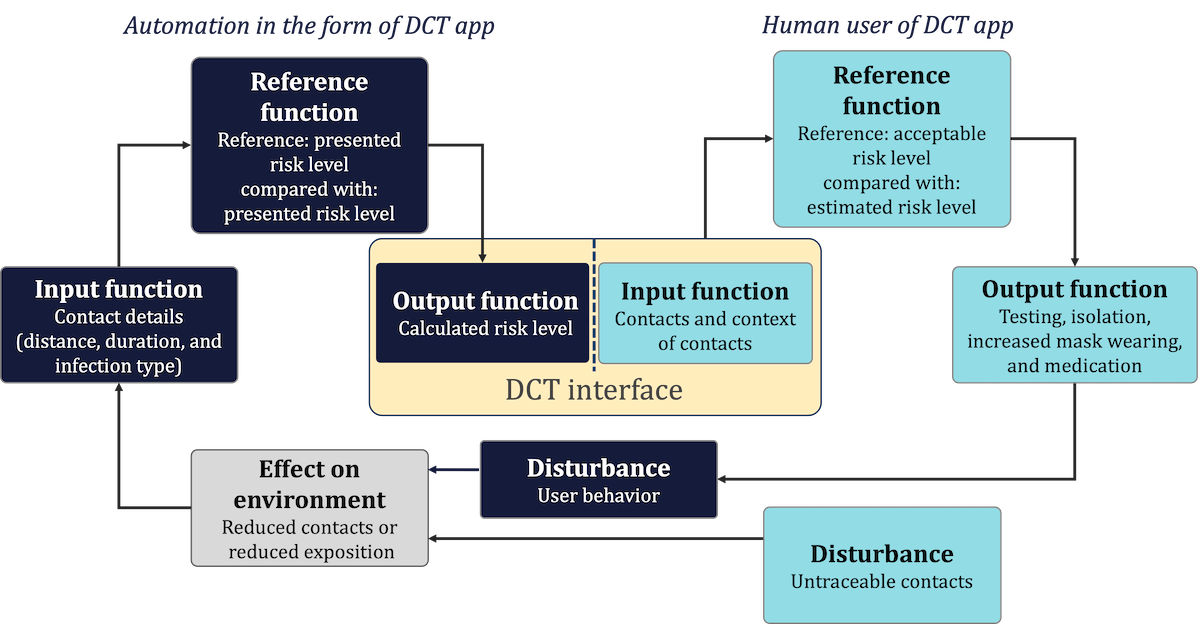
Conceptual control loop model of joint human-machine action regulation in digital contact tracing (DCT). The assessment of the machine processing steps (input, reference, and output) is central to the perceived trustworthiness (perceived data validity, perceived goal congruity, and perceived result diagnosticity) of the system.

Contact tracing involves data gathering but also decision-making processes that influence individual and collective health outcomes. It integrates continuous information processing and, therefore, can be viewed through the theoretical lens of control-theoretical conceptions of human-machine systems. The control loop model of action regulation in contact tracing can be extended to accommodate for DCT as automation (ie, a system) that takes over tasks in the acquisition, analysis, and decision selection of contact information [20]. However, maintaining an acceptable risk level [40] is not a singular, finite process but a continuous one. Accordingly, we propose to model information acquisition, analysis, and decision selection as parts of an action regulation consisting of an input function, a reference function, and an output function. As depicted in Figure 1, both human and machine information processing can be modeled within a conceptual control loop to reflect continuous information processing. The conceptual control loop model (Figure 1) illustrates the integration of human and automation activities into a joint action regulation.

Based on the model presented in Figure 1, we assumed that users’ interaction with DCT apps is based on their evaluation of automated input, reference, and output functions. They assess the correctness of the data that the DCT system uses (*input function*), the data’s congruence with the users’ goals (*reference function*), and the utility of the data’s communicated results (*output function*). Any lack of transparency in their joint action regulation can diminish perceived trustworthiness as well as hamper situation awareness. For instance, if the system fails to capture necessary data accurately or align with personal goals such as identifying the source of infection versus alerting those potentially infected, perceived trustworthiness may decline. Accordingly, parallel to similar phenomena in other automation contexts that do not reveal which information is used as part of the input function, an out-of-the-loop unfamiliarity might cause decreasing situation awareness [20]. Furthermore, the user experience may suffer if the system’s output, such as an imprecise infection risk description, is insufficient for users to decide the next course of action, therefore impeding the perceived usefulness.

In addition, users’ perception of the system is dependent on their expectations of information processing (cf [41]; ie, how the DCT system processes contact-related data). For example, whether a DCT app processes others’ vaccination status will only matter to users who are interested in that information, and disclosing that the app processes vaccination information will only impact the system perception of those users. As such, to understand the formation of perceived usefulness, users’ subjective situation awareness is more important than their factual situation awareness. However, as introduced by Schrills and Franke [42], subjective evaluation of a user’s ability to “perceive, understand and predict a system’s information processing,” described as subjective information processing awareness, can serve as a construct to assess users’ perception of an automation’s effect on situation awareness. However, users’ perception of their information processing awareness might not be reflected in the accuracy of their knowledge about the system’s information processing.

**Figure 2:**
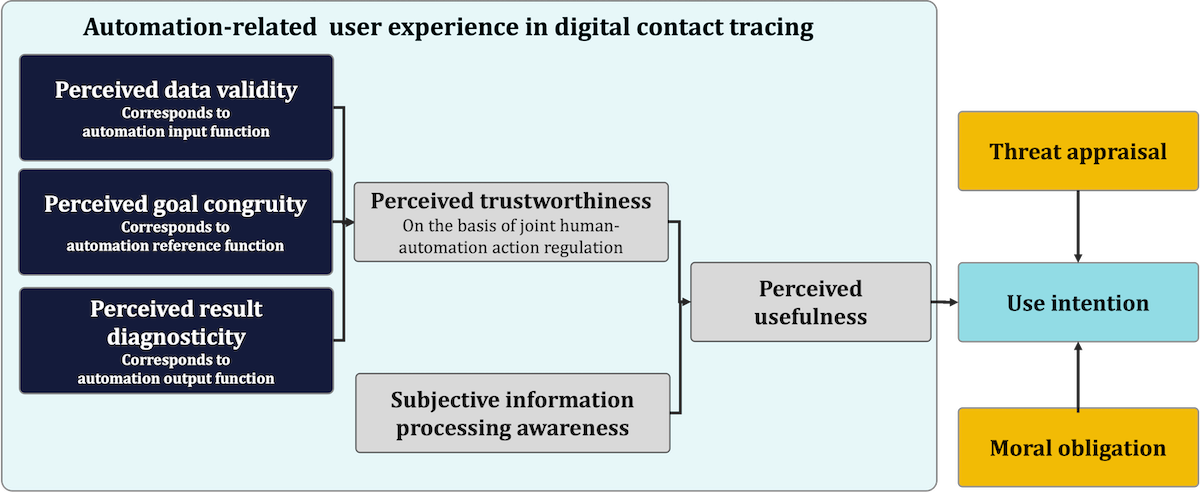
Research model on automation-related user experience and the effect on use intention of digital contact-tracing apps.

The previous concepts of perceived data validity, goal congruity, result diagnosticity, trustworthiness, subjective information processing awareness, and perceived usefulness can be subsumed as automation-related user experience. Automation-related user experience, following the 9241 standard from the International Organization for Standardization, can be defined as *the perception and response of a person resulting from using or anticipating the use of automated systems*. On the basis of our proposed conception of automation-related user experience, we conceptualized a model of factors of use intention in DCT centered on perceived usefulness of automation as depicted in Figure 2. In addition, threat appraisal and moral obligation as factors independent of DCT use are integrated as measures to evaluate the influence of automation-related user experience on use intention comparatively. Threat appraisal and moral obligation are not connected with properties of the DCT app; that is, they influence whether a user wants to demonstrate behavior to trace contacts but not how useful a specific app is perceived to be.

### This Study

On the basis of the presented research model, the objective of this study was to investigate how automation-related user experience affects the perceived usefulness of contact tracing as well as the use intention of DCT and how user experience could be improved. We aimed to contribute to research on DCT adoption and use by examining possible pathways to enhance use intention via user experience. On the basis of the proposed research model, we analyzed the following hypotheses: (1) perceived trustworthiness correlates positively with perceived usefulness (hypothesis 1), (2) subjective information processing awareness correlates positively with perceived usefulness (hypothesis 2), and (3) perceived usefulness correlates positively with use intention (hypothesis 3).

In addition, we examined the relationship among all the aforementioned variables in a structural equation modeling (SEM), where we tested automation-related variables as well as variables not related to the specific DCT system: (1) threat appraisal is positively related to use intention (hypothesis 4) and (2) moral obligation is positively related to use intention (hypothesis 5).

Accordingly, the research model depicted in Figure 2 serves as a basis for an SEM analysis that integrated both automation-related user experience and automation-independent variables (threat appraisal and moral obligation).

We supplemented our quantitative findings with qualitative data on the requirements for improved information processing, providing a deeper insight into users’ interactions with the app. This mixed methods approach allowed us to uncover underlying patterns and themes that cannot be identified through quantitative data alone, providing a more comprehensive understanding of the user experience.

## Methods

### Participants

Participants were recruited via social networks (Twitter [subsequently rebranded X] and Facebook), where an image and a link to the study were shared showing a picture of the CWA and asking for participation (ie, our sample was self-selected). The recruitment strategy specifically targeted individuals who had experience using the CWA. Eligibility for the study required participants to be aged ≥18 years and have at least fluent German skills. The study was conducted on the web, with data collection taking place via a web-based questionnaire between June 1, 2022, and July 31, 2022, using LimeSurvey (LimeSurvey GmbH) [43]. We decided not to inquire further about demographic variables to maintain high levels of privacy due to the context of the study (tracking apps).

A total of 317 participants were included in the study (refer to the Data Exclusion section for further details). As user diversity can have a significant impact on the individual user experience and the perceived trustworthiness, we assessed the affinity for technology interaction (ATI) [44]. ATI describes the individual tendency to actively engage in intensive technology interaction. The ATI was measured using a scale validated in various large samples. Our sample ranged from 1 to 6, with an average value of 4.19 (SD 1.26) which was somewhat higher than the value of 3.5 that Franke et al [44] assumed for the general population based on quota sampling. This corresponds with the self-selection of the sample; we can assume that users who installed the CWA may have, in general, a higher level of ATI than the general population.

### Ethical Considerations

This study was registered (under 2022-413) at the Ethics Committee of the University of Lübeck. Before participating in the study, individuals received detailed information about the study and provided written consent to partake. For anonymity, no additional demographic data of the users were queried. No financial remuneration was provided for participation.

### Scales and Procedure

#### Overview

To capture the psychological concepts described previously, multiple scales were developed and presented to participants after they provided informed consent. Except for those for experienced system traceability [42], all items were generated by the researchers based on theoretical considerations and discussed within a team of 3 experts in human-machine interaction.

All items used a 6-point Likert response scale (*completely disagree*=1, *largely disagree*=2, *slightly disagree*=3, *slightly agree*=4, *largely agree*=5, and *completely agree*=6), with the only exception being the semantic differential used for perceived usefulness. For all variables except knowledge, a mean score of all items of the scale was calculated and used for further analysis. All the original items were in German and are presented in this manuscript in English.

#### Use Intention

Use intention was captured using a 3-item scale focusing on participants’ intention and future commitment to use the CWA during the pandemic (Multimedia Appendix 1).

#### Threat Appraisal

A 4-item scale was used aiming to comprehend the participants’ perceived risk and concerns related to a possible infection (Multimedia Appendix 1).

#### Experienced System Traceability

Experienced system traceability was assessed using the 6-item Subjective Information Processing Awareness scale [42] measuring the perceived transparency, understandability, and predictability of information collection and processing by the system (Multimedia Appendix 1).

#### Moral Obligation

Moral obligation was evaluated using a 3-item scale capturing the participants’ sense of responsibility and ethical obligation toward using the CWA (Multimedia Appendix 1).

#### Perceived Trustworthiness

Perceived trustworthiness was measured across 3 subscales, each addressing the trustworthiness of input, reference, and output in the cybernetic control loop (Multimedia Appendix 1).

#### Perceived Usefulness

Perceived usefulness was assessed using a semantic differential scale with labels indicative of the perceived efficiency, precision, safety, complexity, and reliability of the system when cooperating with it (for instructions and labels, refer to Multimedia Appendix 1).

### Statistical Analysis

#### Overview

The data collected in this study were analyzed using R (version 4.31; R Foundation for Statistical Computing) [45]. Initially, the normal distribution of the data was tested to ensure that assumptions of normality were met. Given that the data did not follow a normal distribution, nonparametric tests such as the Welch 2-tailed *t* test were applied to determine statistical significance. In addition, considering the multiple comparisons performed in the calculation of correlations, a Bonferroni correction was used to control for the risk of type I error. Corrected *P* values are reported. The analysis was based on the preregistration, which can be found under <omitted for blinded review>.

PLS-SEM is a statistical modeling method combining aspects of regression and factor analysis. It allows for the simultaneous estimation of the relationship between indicators (ie, manifest variables) and constructs (ie, the latent variables formed from the manifest variables) and the relationship between the constructs themselves. These parts of the models are called the measurement model and structural model [46]. PLS-SEM is robust to nonparametric data, can work with small samples, and is especially suited for exploratory research [47], making it a great fit for this study. We followed the extensive iterative process of model assessment described in the work by Hair [46]. Our iterative approach is documented in Multimedia Appendix 2.

**Figure 3:**
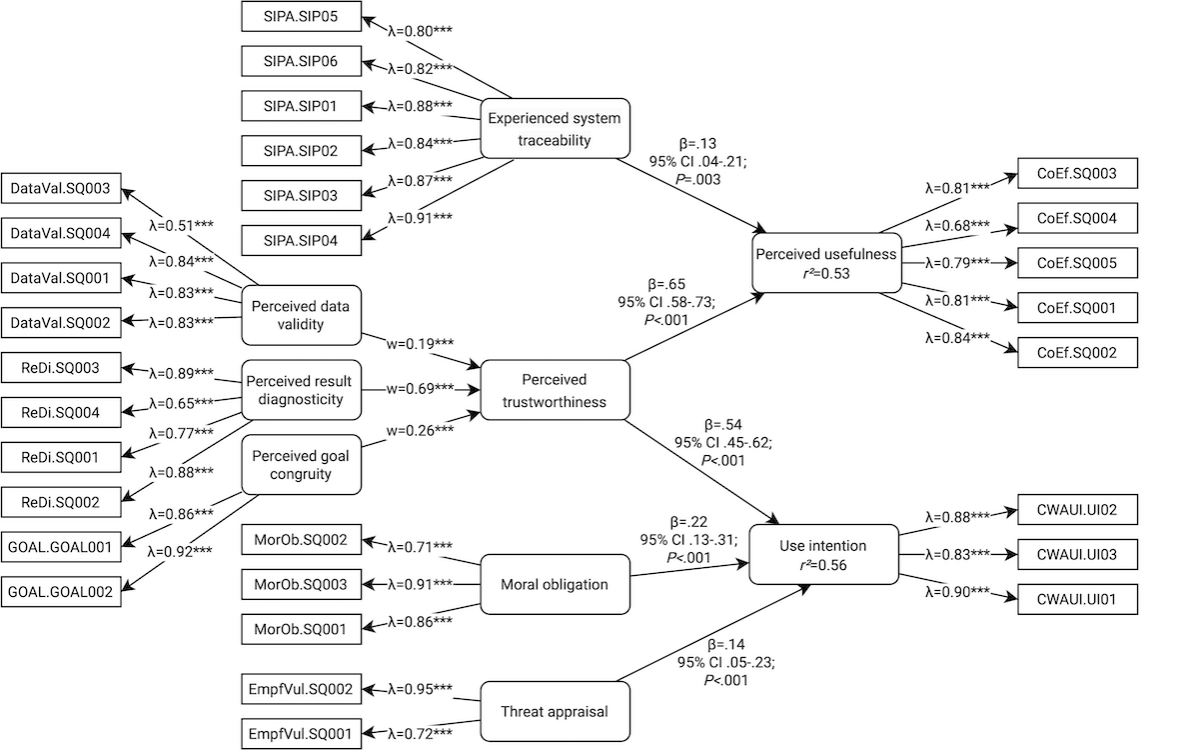
Partial least squares structural equation model after multiple iterations for the proposed research model. Rounded corners indicate constructs based on our research model; rectangular shapes denote indicators that were measured directly in the survey.

The hypothesized PLS-SEM contains all paths depicted in Figure 3. In addition, we tested whether the paths from perceived trustworthiness, system knowledge, and experienced system traceability to use intention were all mediated by perceived usefulness or whether there were also direct effects.

All constructs except perceived trustworthiness were specified as mode-A constructs. The respective indicators are described in the Scales and Procedure section. Perceived trustworthiness was specified as a mode-B higher-order construct consisting of perceived data validity, perceived result diagnosticity, and perceived goal congruity. We report explained variance using *R*^2^, path coefficients using β with *P* values and 95% CIs, and effect sizes using the Cohen *f*^2^.

#### Power

For the PLS-SEM, a retrospective power analysis using the inverse square root method revealed that, given our sample size (N=317), the smallest path coefficient, and a 5% significance level, we achieved a statistical power of 72% [48].

#### Data Exclusion

Before the statistical analysis, the data set with 370 responses was carefully reviewed for any inconsistencies, missing data, and outliers. Cases with incomplete or implausible responses (53/370, 14.3% in total) were identified and excluded from the analysis to maintain the integrity of the data set.

### Qualitative Data Analysis

To obtain a deeper insight into users’ demand for information provision and preservation in the interaction with the CWA, qualitative data were collected via open-ended questions (ie, *what information would you like to get from the system?* [Automation to human; question 1] and *What information would you like to feed to the system?* [Human to automation; question 2]).

As a widely used tool, thematic analysis aims to support the systematic identification, analysis, and reporting of patterns (ie, themes) in qualitative reporting data. Both inductive and deductive approaches were applied using theoretical assumptions as the basis for creating the themes, which were then adapted based on the data collected [49]. The data were coded using MAXQDA (version 20; VERBI GmbH [50]). For a structured and reliable analysis approach, a coding scheme with clear definitions of codes and example coding was developed in multiple iterations (Multimedia Appendix 3). For the evaluation, two perspectives of information needs between humans and automation should be covered: (1) human to automation and (2) automation to human. In total, 2 coders coded the data based on the developed scheme. An intercoder reliability of κ=0.90 (for automation-to-human information demands) and κ=0.87 (for human-to-automation information demands) was achieved. Hence, the level of agreement was strong in both cases [51].

Coded themes for information needs in both automation to human and human to automation included contact or risk information, pandemic-related information, app-related information, and assumptions for perceived information processing. Subcodes were created to enhance coding accuracy (Multimedia Appendix 3) but were not analyzed in detail as the focus remained on the top-level codes. Codes that could not be assigned to one of the themes were assigned to the category *others*. As several participants commented, for example, on the suspected reasons for the limitation of information processing, another category was added (ie, assumed reasons for perceived information processing) to avoid losing these data. Both the categories *others* and *assumed reasons for perceived information processing* were not evaluated for this study.

Missing answers to the questions asked and specific statements that there was no demand for information were assigned the code *none*. This code was assigned only once per person and statement. Thus, in the end, it was possible to clearly distinguish how many of the 317 respondents indicated information needs and how many did not. Ultimately, automation-to-human information demand statements from 45.4% (144/317) of the participants and human-to-automation information demand statements from 27.1% (86/317) of the participants were analyzed (Table 1).

**Table 1:** Number of respondents that indicated information demands versus no information demands.

Variable			Response distribution, n (%)		
			Respondents (n=317)		Responses (n=377)
**Demands**					
	Information demand (A2H^a^)	144 (45.4)		257 (68.2)	
	Information demand (H2A^b^)	86 (27.1)		120 (31.8)	
**No demands**					
	Information demand (A2H)	173 (54.5)		120 (31.8)	
	Information demand (H2A)	231 (72.9)		257 (68.2)	

^a^A2H: automation to human.

^b^H2A: human to automation.

## Results

### Overview

For hypothesis 1, the analysis revealed moderate positive correlations for all factors of perceived trustworthiness. The correlation between data validity and perceived usefulness was significant, with a coefficient of *r*=0.38 and *P*<.001. The correlation between goal congruity and perceived usefulness showed a coefficient of *r*=0.47 and *P*<.001, indicating a moderate positive linear relationship. Result diagnosticity and perceived usefulness exhibited a strong positive correlation, with a coefficient of *r*=0.56 and *P*<.001. In general, all measures of perceived trustworthiness and perceived usefulness exhibited a positive relationship, supporting hypothesis 1.

For hypothesis 2, a correlation coefficient of *r*=0.35 (*P*<.001) was observed, suggesting a moderate positive linear relationship between subjective information processing awareness and perceived usefulness; a positive relationship between SIPA and perceived usefulness (hypothesis 2) was supported by the data. This indicates that automation-related phenomena such as changes in situation awareness might influence the perceived usefulness of DCT.

For hypothesis 3, the correlation coefficient between perceived usefulness and use intention was *r*=0.47 and *P*<.001, indicating a moderate positive correlation. Hence, our results support the hypothesis (hypothesis 3) that perceived usefulness is positively related to use intention (hypothesis 3). In combination with our previous results, this indicates strong relationships between user experience variables and use intention.

In summary, all variables showed statistically significant correlations with perceived usefulness. These correlations ranged from moderate to strong positive relationships. These results strengthen our assumption that perceived usefulness of DCT is strongly related to automation-related user experience.

### SEM Approach

The final PLS-SEM is depicted in Figure 3. The explained variance for use intention was *R*^2^=0.56. It was directly predicted by perceived trustworthiness (β=.54, 95% CI .45-.62; *P*<.001; *f*^2^=0.44), moral obligation (β=.22, 95% CI .13-.31; *P*<.001; *f*^2^=0.07), and threat appraisal (β=.14, 95% CI .05-.23; *P*<.001; *f*^2^=0.04). Thus, there was a large effect for perceived trustworthiness and a small effect for the other constructs. Still, hypotheses 4 and 5 were supported.

Within the perceived trustworthiness higher-order construct, the highest weight was assigned to perceived result diagnosticity (*w*=0.69; *P*<.001), implying that this subconstruct contributes most to perceived trustworthiness, followed by perceived goal congruity (*w*=0.26; *P*<.001) and perceived data validity (*w*=0.19; *P*<.001).

We did not find evidence for a mediating effect of perceived usefulness on the paths from perceived trustworthiness, system knowledge, and experienced system traceability to use intention. However, we did find direct effects of perceived trustworthiness (β=.65, 95% CI .58-.73; *P*<.001; *f*^2^=0.65) and experienced system traceability (β=.13, .04-.21; *P*=.003; *f*^2^=0.02) on perceived usefulness (*R*^2^=0.53).

### Qualitative Analysis

#### Overview

Two directions of information flow were analyzed to assess the information demands of CWA users: (1) human to automation—information that users want to provide to the system and (2) automation to human—information that users want to receive from the system. In total, 3 overarching themes were explored and analyzed in more detail (Textbox 1).

**Analyzed themes and description of each theme. The detailed coding scheme can be found in Multimedia Appendix 3.**

**Contact- or risk-related information**

- Time-related information: information regarding the period of the contact, the duration of the contact, the time passed since the contact, and the period during which contact tracing was possible
- Location-related information: information related to the place of contact, direct or indirect contact, and indoor or outdoor contact
- Exposition-related information: information about the masking status in the contact situation and the distance between the persons in contact
- Action-related information: information on possible and suggested courses of action after contact
- Information related to the warning person: information concerning the time when the warning person tested positive, the time when the warning person became infected, the warning person’s first symptoms, the warning person’s vaccination status, and the infected person’s virus variant

**Pandemic-related information**

- Statistics: information related to statistical content on the pandemic in terms of the number of defects or infections

**App-related information**

- Number of users: information about the number of users of the Corona-Warn-App
- General calculation-related information: information on reasons for changing risk calculation and the system parameters used for calculations
- Certainty about the result: information related to the certainty of the results calculated by the system
- Integration of tests (self- and externally administered): information about the possibility to enter or delete test results on the app
- Linking with private data: information on the possibility of linking app functions with private data

#### Descriptive Data

##### Overview

The overall number of statements amounted to 211 in automation to human and 76 in human to automation. Within these 2 categories, the themes were distributed unevenly. Information regarding contact and risk accounted for most statements in both categories (automation to human: 196/211, 92.9% of statements; human to automation: 62/76, 82% of statements). The remaining statements were (almost) exclusively distributed among app-related information (automation to human: 14/211, 6.6% of statements; human to automation: 14/76, 18% of statements) as barely any needs were stated for pandemic-related information (automation to human: 1/211, 0.5% of statements; human to automation: 0 statements).

**Figure 4:**
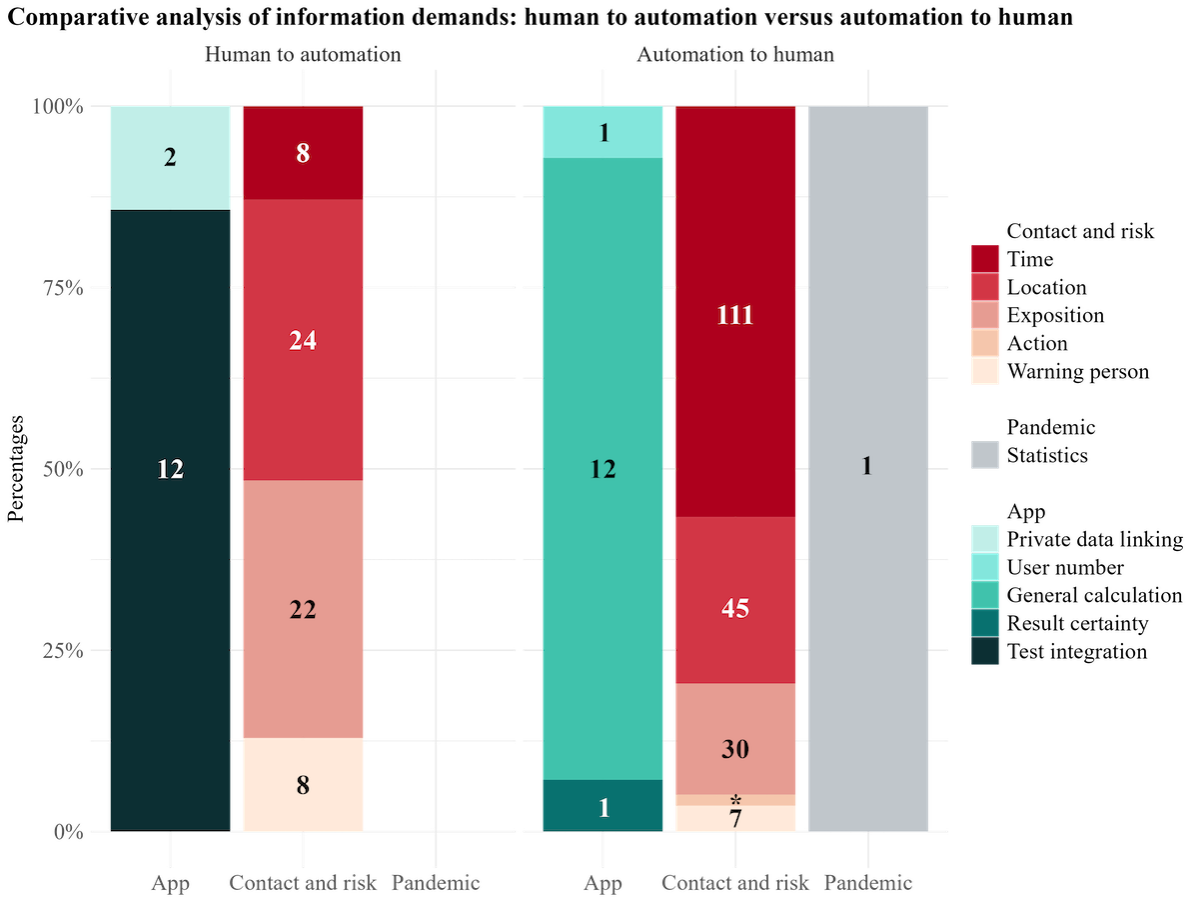
Relative demands regarding information from automation to human (left) and from human to automation (right). The numbers in the column sections indicate the number of statements under each code.

Regarding the subcodes, the distribution also varied between both themes (Figure 4). For contact- and risk-related information, the information related to time, location, and exposure accounted for the largest proportion of demands within this theme in both categories. However, the distribution of statement proportions differed clearly between automation to human and human to automation. Time-related information was demanded most in automation to human (111/196, 56.6% of statements) but least in human to automation (8/62, 13% of statements). Demands for location-related information did not differ greatly between automation to human (45/196, 23% of statements) and human to automation (24/62, 39% of statements), nor did exposition-related information (automation to human: 30/196, 15.3% of statements; human to automation: 22/62, 35% of statements).

In terms of *app-related information*, the demands for information about the system’s general calculation (automation to human: 12/14, 86% of statements; human to automation: 0% of statements) and the integration of tests (automation to human: 0% of statements; human to automation: 12/14, 86% of statements) differed in particular between the categories. The remaining subcodes hardly received any consideration. In both categories (automation to human and human to automation), almost no statements regarding *pandemic-related information* were made.

##### Human to Automation

In human to automation, certain claims emerged with particular frequency in the demand for contact- and risk-related and app-related information. Information demands on contact and risk mainly focused on time*-* and exposure-related information. For example, the interest in informing the app of one’s location and whether one was in an enclosed space or outdoors was present:

Tell the app something about the specific location (enclosed space, fresh air).

Exposition-related information demands mainly focused on informing the app when one wore or had worn a mask:

The wearing of a mouth-nose covering should be entered and thus taken into account in the risk calculation.

Regarding the demand for the integration of app-related information, the participants predominantly highlighted the integration of self-administered or externally administered tests:

That I am Corona positive without having done a Polymera-Chain Reaction (PCR) test. (Perhaps with indication that the result is not PCR verified).

##### Automation to Human

In the automation to human category, contact- and risk-related and app-related information were queried with similar frequency. The contact- and risk-related information in this category most often referred to time-related information with a request for the time of the risk encounter. However, the desired preciseness of the temporal data differed (exact time vs more approximate time: “When was the encounter? (At least as a time frame, e.g., between 8-12 o'clock)” vs “The specific time [...] of a risk encounter would be helpful”). The location of the risk encounter was another type of information that participants commonly solicited. Most asked for information about a rather specific location (“At which location did a contact take place?”); few seemed to be interested in the characteristics of the location (“Indoors or outdoors?”).

Exposure-related information demanded from the system included the number of devices or persons present at the time of exposure (“[...] with how many devices was the contact?”), the distance to the warning person (“At what distance was the encounter?”), and the masking status. In particular, masking status included the person’s own status of having worn a mask or whether the other person was wearing a mask at the time of the risk encounter (“Was I wearing a mask? Was the other person wearing a mask?”).

App-related information demands mainly focused on the parameters of the calculation (“What factors led to this result?”) and reasons for a status change (“How exactly the risk determination works, i.e., how distance and time to a positively tested person actually have to be, in order for me to receive a notification and for the status to be changed”).

## Discussion

### Principal Findings

The objective of this study was to understand automation-related user experience, its connection to perceived usefulness, and the use intention of DCT. Our data showed that perceived trustworthiness is a critical factor in understanding use intention as well as the perceived usefulness of DCT apps. Interestingly, users’ experience of a system as supportive in their action regulation affects their use intention more strongly than external factors such as threat appraisal or moral obligation. In addition, our qualitative analysis revealed that users mainly want to communicate with the system about information that is relevant to their decision-making. For instance, providing more precise information about masking status when in contact with other people could assist a user in making an immediate decision regarding isolation. Overall, our findings suggest a strong relationship between the diagnosticity of automated information processing and use intention.

### Practical Implications

As a first major implication, the high effect of result diagnosticity on perceived trustworthiness demonstrates the importance of human-centered information processing in (partially) automated health applications. Within the interconnected human-machine information processing loops (Figure 1), the machine provides information as part of the human input function. As discussed by Miller [52], intelligent systems such as DCT should aim to improve users’ ability to access and use (processed) information rather than to present and justify a particular outcome. In DCT app design, the integration of DCT information into a joint human-automation action regulation should be prioritized. Accordingly, when developing evaluative systems [52] that support the evaluation of alternatives rather than suggesting specific actions, it is important to consider what evaluative process a user needs to undertake. While previous research has already identified the need for actionable information [53], the information presented by DCT apps needs to be understood in the context of human action regulation and the influence of automated systems in human action regulation. A possible solution to support diagnosticity in DCT is so-called proactive contact tracing [54], which integrates more information sources and can potentially enrich DCT results.

Second, the results indicate a strong user need for information to be provided in sufficient detail. An interface optimized for communicating information could enable users to make their own assessment of the situation. In many DCT apps, users request the ability to retrieve information about possible contacts, such as time, location, or even the person involved [55]. Our study showed similar results (eg, a high demand for detailed information about the [exact] time of detected contacts). Again, the demand for more detailed information relates to the diagnosticity of the information provided by the system. If users are only given information about their current risk of infection, they cannot evaluate the validity of this information, potentially leading them to ignore it. They would require additional context-related information about potential contacts, such as whether the individuals were wearing masks or were located in an enclosed room, to make informed decisions about their behavior. Our results demonstrate that use intention is strongly connected to the perceived diagnosticity of the DCT app. On the basis of our qualitative findings, we can assume that the diagnosticity of DCT users depends on the level of detail they receive about possible contacts. Accordingly, the provision of details that support users’ information processing is even more important for their use intention than threat appraisal or moral obligation. In accordance with psychological research on motivation [56], supporting users’ intrinsic motivation for diagnostic information could lead to better adherence regarding DCT apps than, for instance, exposing them to extrinsic motivators that increase threat appraisal (eg, describing the consequences of infection [57]).

Third, in contradiction to users’ demand for detailed information on contacts, a major concern in DCT is privacy [55]. While it is often argued that too much detail conflicts with privacy, it is important to find ways to improve the diagnosticity of information as this determines the use intention. Possible solutions include differential privacy, which allows for sufficient detail for increased diagnosticity while keeping personal data confidential. In addition, many users requested features that do not compromise the privacy of others, such as the ability to inform the system about masking status. Thus, allowing users to refine the input received by the DCT app may increase the perceived diagnosticity of the results. The integration of masking status can be seen as a measure to improve the accuracy of the apps in determining risk levels, ultimately increasing the use intention.

Overall, our results suggest that focusing on the diagnosticity of the information presented in DCT apps could result in improvement in users’ health behavior. During the COVID-19 pandemic, users reported that they were unsure about the correct or best action to take to contain the pandemic or could not correctly assess the risk of certain situations [58]. However, this certainty is particularly important when it comes to health decisions. With sufficient diagnostic accuracy, DCT apps may be able to better reduce this uncertainty and, thus, become a crucial component in the management of pandemics in the long term, also positively affecting users’ willingness to provide data on a social level. It is also crucial that DCT apps do not follow the *recommend and defend* principle [52], which could lead to a long-term reduction in motivation, but instead provide information that supports individual decisions. If compliance with effective pandemic control measures can be increased as a result, it will be possible to respond more effectively to future pandemics.

### Theoretical and Methodological Implications

In our data, the perceived trustworthiness of a DCT app had a greater influence on use intention than threat appraisal or moral obligation. Furthermore, while previous studies [26] have relied on perceived usefulness, our findings in the PLS-SEM do not suggest that it mediates the relationship between perceived trustworthiness and use intention. However, usefulness can be seen as an ambiguous concept without a specific connection to the design of DCT apps. In this way, focusing on perceived usefulness could hinder approaches to improve DCT by adopting DCT app design and functionality. In contrast, a lack of perceived result diagnosticity indicates to developers that the information provided by a DCT app needs to be adapted to have an impact on joint action regulation. Our research suggests that designers of automated systems should specify the potential actions that users can take and identify decision points at which users may require diagnostic information, such as whether to proceed with a specific action. In addition, highlighting the role of diagnosticity indicates how models of technology in medical systems should be developed. Existing models (such as the technology acceptance model) do not specify to what extent a system’s usefulness depends on perceived diagnosticity. Our research demonstrates that behavioral models focusing on information-based decisions are needed to address automated technology in health, for example, DCT.

However, one can argue that the difference between perceived result diagnosticity and perceived usefulness is arbitrary; in a joint human-automation action regulation, the diagnosticity of information seems to be equal to perceived usefulness. However, by directly addressing perceived result diagnosticity as a central variable of automation-related user experience, empirical research can identify paths to improve action regulation support of DCT without previously defining what is useful about a system or not. When a DCT app can deliver information that users can use to regulate their actions, users report a higher intention to use it. Therefore, applying result diagnosticity as a variable in human-automation research is a methodological contribution supporting future research in intelligent automation.

On the basis of our findings, future research on DCT needs to determine how to improve the diagnosticity of DCT apps. This paper introduced a conceptual control loop model of joint human-machine action regulation, which can support research approaches in optimizing perceived diagnosticity as a central variable for automation-related user experience. Addressing the joint action regulation in DCT and health behavior is crucial to understand how the information provided by DCT apps can be integrated into human information processing and how DCT apps influence the human output function. Information that improves the evaluation of individual contacts, such as contact location, masking status, or vaccination level, could improve perceived trustworthiness and use intention of DCT apps. By demonstrating how information processing between human users and DCT apps is integrated, our research supports a shift from viewing human users as receivers of machine results to viewing them as actors using DCT information.

All in all, our findings regarding the significance of diagnosticity have implications for the design of automated information processing in a broader context. Users did not primarily prioritize data validity or goal congruence; instead, their focus lay in determining whether they could trust the system to provide information that would assist their own decision-making process. This may be a general trend in automated information processing.

### Limitations and Further Research

All participants of this study were users of the CWA. However, as Walrave et al [59] describe, many citizens in Germany did not use DCT apps, for example, because they did not want to share their data or did not think they were effective. Thus, the findings presented on the impact of perceived diagnosticity may not be applicable to citizens who did not use the app at all. These individuals may have chosen not to use the app for reasons beyond those discussed in this paper. The perceived diagnosticity of a DCT app is only relevant for use intention when potential users are interested in determining their individual risk level or making decisions based on their estimated risk level. That is, our sample may bias the results and underestimate factors relevant to nonusers. For example, nonusers might reject the app because they do not trust the provider of the system. Accordingly, the results of our study may support improving DCT for existing users but not convincing nonusers to use DCT. Further studies need to address nonusers and examine how automation-related user experience affects their decision not to use DCT.

In addition, users may have misconceptions about the factors contributing to the risk of infection and may expect the system to provide irrelevant information that does not aid in making an informed decision. Accordingly, they might report a low perceived diagnosticity while the information provided in the app offers sufficient diagnosticity. The accuracy of one’s mental model [60] may influence the perception of actual diagnostic information as nondiagnostic (for a discussion of diagnosticity, refer to the study by Garcia-Marques et al [61]). To tackle false models of diagnosticity, DCT apps should support users in correcting their mental model, for example, by explaining how they can use the provided information. This could be done by simulating decision situations with and without DCT information, offering users the experience of diagnosticity.

Improving the perceived diagnosticity could be beneficial for use intention but could negatively affect perceived data privacy [55]. For example, a function that allows users to communicate when they are wearing a mask could be abused to track specific contacts, therefore revealing potential infections of other users. Data privacy is a critical concern in DCT use [59]. Therefore, current DCT apps are designed to protect the data of other users at the cost of the diagnosticity of information. This research did aim to understand the effect of user experience in automated DCT but did not include how users evaluate potential risks of data privacy violations or approaches to address them (cf [62]). Future research should identify how to balance the desired level of perceived result diagnosticity and data privacy concerns. For example, in direct communication, users who reveal information about their web-based status can see the web-based status of others, allowing them to choose which balance between diagnosticity and data protection they desire. The same function could be implemented in DCT apps to support automation-related user experience. Allowing users to choose their level of diagnosticity themselves allows them also to control how DCT apps influence their decision-making, thus strengthening user autonomy.

Finally, this study had a cross-sectional design that did not assess how automation-related user experience and use intention regarding a DCT app may change over time. Previous research has demonstrated that automation-related user experience can change over time (eg, because users adapt to the system or they improve how they use the system). Future research on automation-related user experience in DCT apps needs to include a longitudinal study design to capture effects of behavior change and users’ perception.

### Conclusions

In conclusion, this research highlights the relevance of automation-related user experience in DCT and its role in enabling the effective action regulation of DCT users. Here, providing detailed and diagnostic information is crucial for users to make informed assessments of their situation and actions. The presented quantitative results echo the qualitatively assessed user demand for more detailed information about potential contacts, such as time, location, and context (eg, mask use and indoor or outdoor setting).

Interestingly, our data suggest that other factors not directly related to the app, such as moral obligation and threat appraisal, are less relevant compared to automation-related user experience, especially to the perceived diagnosticity of the information provided by DCT apps. The presented results are also more specific than those of previous studies that relied on perceived usefulness. Our research model did not suggest that perceived usefulness mediates the relationship between perceived trustworthiness and use intention. Instead, we propose that DCT designers should focus on providing diagnostic information at critical decision points.

However, privacy remains a major concern in DCT. While it is often argued that too much detail conflicts with privacy, it is crucial to find ways to improve the diagnosticity of information without compromising privacy. Solutions could include differential privacy or features that do not compromise the privacy of others, such as the ability to inform the system about masking status.

The main impact of our results on the design of DCT apps and health policy is that DCT apps need to provide sufficient diagnosticity to be perceived as useful. This means that (1) the possible actions of users need to be understood before the design of the DCT algorithm and apps and (2) the presented information needs to support them in choosing the correct action. Focusing on the diagnosticity of the information presented in DCT apps could, in turn, also influence user performance. During the COVID-19 pandemic, a significant percentage of users reported uncertainty about the best actions to take or could not correctly assess the risk of certain decisions. Therefore, improving diagnostics could contribute to better and safer decisions.

In summary, our study underscores the importance of balancing detailed and diagnostic information with privacy concerns in DCT apps. As we move forward in this digital age, it is crucial to continue exploring ways to optimize DCT while respecting user privacy.

## Appendix

Multimedia Appendix 1 Scales for the cross-sectional survey study on user experience.

Multimedia Appendix 2 Documentation of the iterative process of partial least squares structural equation modeling.

Multimedia Appendix 3 Coding scheme.

## Abbreviations

ATI: affinity for technology interaction
CWA: Corona-Warn-App
DCT: digital contact tracing
HBM: Health Belief Model
PLS-SEM: partial least squares structural equation model
SEM: structural equation modeling
