# Supplementary material for: Effects of User Experience in Automated Information Processing on Perceived Usefulness of Digital Contact-Tracing Apps: Cross-Sectional Survey Study
Source: JMIR Hum Factors. 2024 Jun 25;11:e53940. doi: 10.2196/53940 (PMC11234054; doi:10.2196/53940)
Supplement: Multimedia Appendix 1 [file humanfactors_v11i1e53940_app1.pdf]

Scales

Scales for Cross-sectional Survey Study on User Experience

All scales were originally in German and are translated. The translation have not been validated. All items use a 6-point Likert response scale from completely disagree = 1, largely disagree = 2, slightly disagree = 3, slightly agree = 4, largely agree = 5, to completely agree = 6

Items marked with * are reversed.

Use Intention

Please indicate the degree to which you agree/disagree with the following statements.						
	1	2	3	4	5	6
The CWA is an important component for me in dealing with the pandemic.						
I will use the CWA to feel safer.						
I plan to follow the behavioral guidance in the CWA.						

Threat Appraisal

Please indicate the degree to which you agree/disagree with the following statements.						
	1	2	3	4	5	6
An undetected infection with the coronavirus has no negative consequences for me. *						
The fact that I can become infected without knowing it worries me.						
It is not important to me to know my risk of infection.						
I don't care if I don't know which contacts to inform after being infected. *						

Moral Obligation

Please indicate the degree to which you agree/disagree with the following statements.						
	1	2	3	4	5	6
I have a moral obligation to use the Corona Warning App.						
I feel responsible for reducing the risk of infection to others during the COVID-19 pandemic.						
We should all use the Corona Warning app for ethical reasons.						

Experienced System Traceability

Please indicate the degree to which you agree/disagree with the following statements.						
	1	2	3	4	5	6
It was transparent to me which information was collected by the system.						
The information that the system could acquire was observable for me.						
It was understandable to me how the collected information led to the result.						
The system's information processing was comprehensible to me.						
With the information accessible for me, the result was foreseeable for me.						
The system's information processing was predictable for me.						

Perceived Data Validity (Perceived Trustworthiness I)

Please indicate the degree to which you agree/disagree with the following statements.						
	1	2	3	4	5	6
The information with which the CWA works is correct.						
The information used by the CWA is complete.						
The CWA does not have access to all relevant information.						
The information used by the CWA is inaccurate. *						

Perceived Goal Congruency (Perceived Trustworthiness II)

Please indicate the degree to which you agree/disagree with the following statements.						
	1	2	3	4	5	6
The goals of the CWA are in line with my goals.						
Supporting the CWA in its actions benefits myself.						
Using the CWA is detrimental to myself. *						
The CWA evaluates information using inappropriate criteria or comparisons. *						

Perceived Result Diagnosticity (Perceived Trustworthiness III)

Please indicate the degree to which you agree/disagree with the following statements.						
	1	2	3	4	5	6
The results of the CWA are not meaningful. *						
When the CWA shows me information, it makes my decisions easier.						
I can do something with the results of the CWA.						
I cannot assess what role the results of the CWA play for me. *						

Perceived Usefulness

By cooperating with the system, the processing of information in tracking contacts and assessing my overall risk status:							
	1	2	3	4	5	6	
faster							slower
More precise							Less precise
Safer							Less safe
More reliable							Less reliabe
