# Supplementary material for: Effects of User Experience in Automated Information Processing on Perceived Usefulness of Digital Contact-Tracing Apps: Cross-Sectional Survey Study
Source: JMIR Hum Factors. 2024 Jun 25;11:e53940. doi: 10.2196/53940 (PMC11234054; doi:10.2196/53940)
Supplement: Multimedia Appendix 2 [file humanfactors_v11i1e53940_app2.pdf]

Effects of User Experience in Automated Information Processing Systems on Perceived Benefits of Digital Contact Tracing Applications: PLS-SEM Evaluation and Iteration

Anonymized

2023-10-24

Contents

1 Data check **2**

2 Iteration 1 Measurement model evaluation **2**
2.1 Model plots . . . 2
2.2 Evaluation of the mode A measurement model . . . 2
2.3 Evaluation of the mode B measurement model . . . 6
2.4 Evaluation of the mode B higher-order constructs . . . 6
2.5 Results summary . . . 6

3 Iteration 2 Measurement Model Evaluation **6**
3.1 Model plots . . . 6
3.2 Evaluation of the mode A measurement model . . . 6
3.3 Evaluation of the mode B measurement model . . . 8
3.4 Evaluation of the mode B higher-order constructs . . . 8
3.5 Results summary . . . 10

4 Iteration 2 Structural Model Evaluation **10**
4.1 Model plots . . . 10
4.2 Collinearity . . . 10
4.3 Mediation analysis . . . 11
4.4 Significance and relevance of path coefficients . . . 11
4.5 In-sample predictive power . . . 11
4.6 Effect size . . . 11
4.7 Summary table . . . 12
4.8 Results summary . . . 12

5 Iteration 3 Structural Model Evaluation **12**
5.1 Model plots . . . 12
5.2 Collinearity . . . 12
5.3 Significance and relevance of path coefficients . . . 12
5.4 In-sample predictive power . . . 14
5.5 Effect size . . . 14
5.6 Summary table . . . 14
5.7 Results summary . . . 14
5.8 Achieved power . . . 14

1 Data check

Before starting with model estimation, we will check the data for excessive skewness and kurtosis. Hair et al. (2017) recommend that manifest variables (indicators) that exhibit skewness or kurtosis > |1| should be excluded from estimation.

Skew and/or kurtosis are problematic (> |1|) for the following variables: [1] "EmpfVul.SQ004" "SIPA.SIP01" "MorOb.SQ001" "MorOb.SQ002" "MorOb.SQ003" "GOAL.GOAL001" "GOAL.GOAL002" [8] "GOAL.GOAL003" "CoEf.SQ001" "CoEf.SQ002" "CoEf.SQ003" "CoEf.SQ005"

If one sets a more liberal interval of +-5, the following variables should be excluded: [1] "GOAL.GOAL003"

2 Iteration 1 Measurement model evaluation

2.1 Model plots

2.1.1 Original Estimate Path Model

This is the original estimate path model.

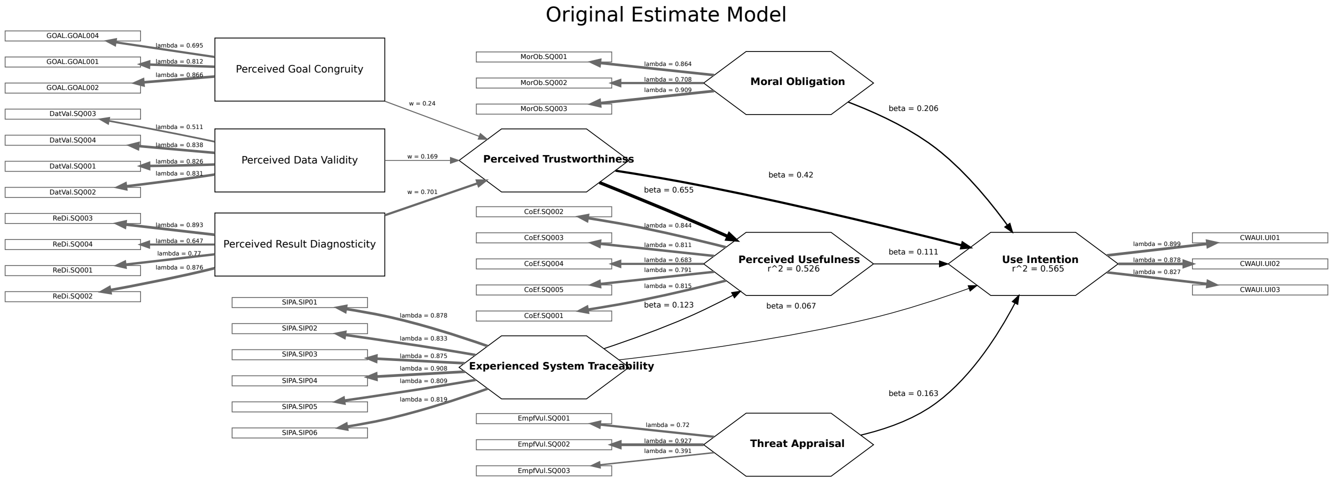

Figure 1: Original estimate model iteration 1

(#fig:plot model)

2.1.2 Bootstrapped Path Model

This is the bootstrapped path model.

2.1.3 Measurement Model Only

This is a path model showing only the measurement model components.

2.2 Evaluation of the mode A measurement model

2.2.1 Convergent validity

Ideally, outer loadings (l) should be ≥ 0.70. Loadings below 0.40 are unacceptable. AVE should be > 0.50.

2.2.2 Internal consistency reliability

Both Cronbach's a and composite reliability rc should be ≥ 0.60 and ≤ 0.90. The upper threshold of acceptability is 0.95.

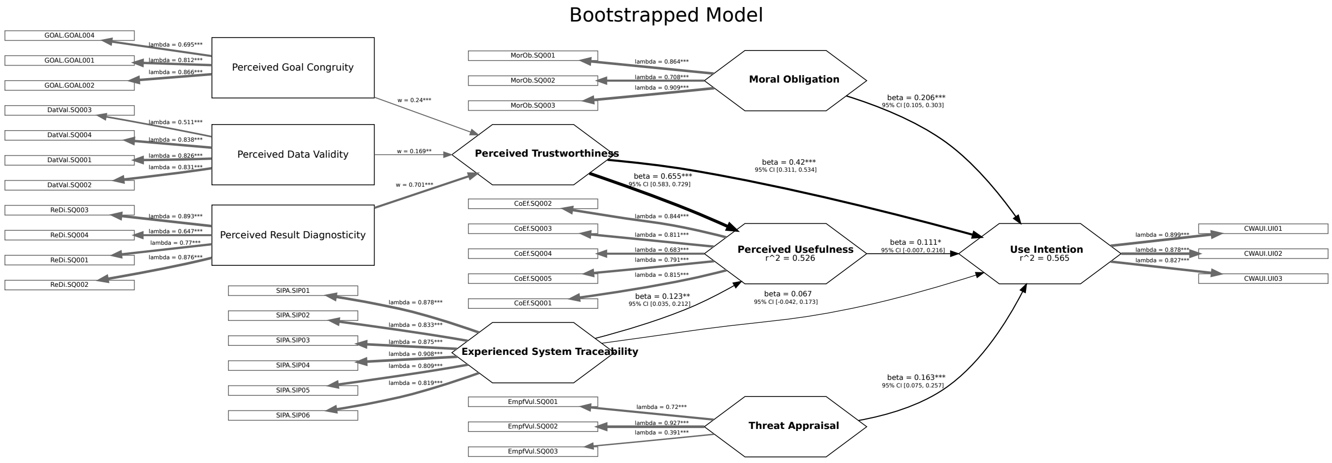

Figure 2: Bootstrapped model iteration 1

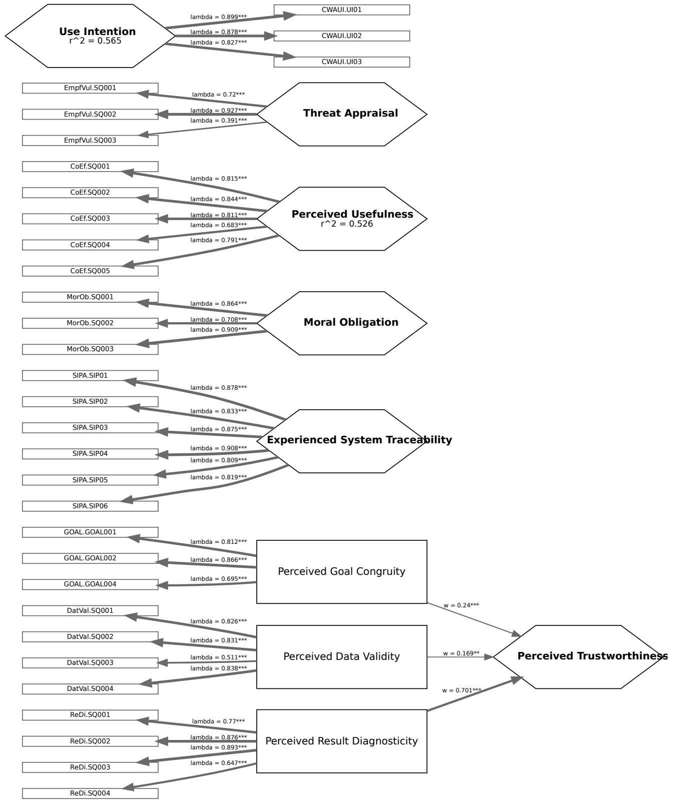

Figure 3: Only measurement model iteration 1

Table 1: Results of the mode A measurement model evaluation model iteration 1

Construct	Indicator	Loading	AVE	Calpha	rhoC	rhoA	1.in.HTMT.CI
Perceived Goal Congruity	GOAL.GOAL001	0.812	0.631	0.706	0.836	0.734	TRUE
Perceived Goal Congruity	GOAL.GOAL002	0.866	0.631	0.706	0.836	0.734	TRUE
Perceived Goal Congruity	GOAL.GOAL004	0.695	0.631	0.706	0.836	0.734	TRUE
Perceived Data Validity	DatVal.SQ001	0.826	0.584	0.759	0.844	0.818	FALSE
Perceived Data Validity	DatVal.SQ002	0.831	0.584	0.759	0.844	0.818	FALSE
Perceived Data Validity	DatVal.SQ003	0.511	0.584	0.759	0.844	0.818	FALSE
Perceived Data Validity	DatVal.SQ004	0.838	0.584	0.759	0.844	0.818	FALSE
Perceived Result Diagnosticity	ReDi.SQ001	0.770	0.644	0.813	0.877	0.852	TRUE
Perceived Result Diagnosticity	ReDi.SQ002	0.876	0.644	0.813	0.877	0.852	TRUE
Perceived Result Diagnosticity	ReDi.SQ003	0.893	0.644	0.813	0.877	0.852	TRUE
Perceived Result Diagnosticity	ReDi.SQ004	0.647	0.644	0.813	0.877	0.852	TRUE
Experienced System Traceability	SIPA.SIP01	0.878	0.730	0.926	0.942	0.928	FALSE
Experienced System Traceability	SIPA.SIP02	0.833	0.730	0.926	0.942	0.928	FALSE
Experienced System Traceability	SIPA.SIP03	0.875	0.730	0.926	0.942	0.928	FALSE
Experienced System Traceability	SIPA.SIP04	0.908	0.730	0.926	0.942	0.928	FALSE
Experienced System Traceability	SIPA.SIP05	0.809	0.730	0.926	0.942	0.928	FALSE
Experienced System Traceability	SIPA.SIP06	0.819	0.730	0.926	0.942	0.928	FALSE
Perceived Usefulness	CoEf.SQ001	0.815	0.625	0.849	0.892	0.855	FALSE
Perceived Usefulness	CoEf.SQ002	0.844	0.625	0.849	0.892	0.855	FALSE
Perceived Usefulness	CoEf.SQ003	0.811	0.625	0.849	0.892	0.855	FALSE
Perceived Usefulness	CoEf.SQ004	0.683	0.625	0.849	0.892	0.855	FALSE
Perceived Usefulness	CoEf.SQ005	0.791	0.625	0.849	0.892	0.855	FALSE
Moral Obligation	MorOb.SQ001	0.864	0.692	0.776	0.870	0.825	FALSE
Moral Obligation	MorOb.SQ002	0.708	0.692	0.776	0.870	0.825	FALSE
Moral Obligation	MorOb.SQ003	0.909	0.692	0.776	0.870	0.825	FALSE
Threat Appraisal	EmpfVul.SQ001	0.720	0.510	0.535	0.739	0.854	FALSE
Threat Appraisal	EmpfVul.SQ002	0.927	0.510	0.535	0.739	0.854	FALSE
Threat Appraisal	EmpfVul.SQ003	0.390	0.510	0.535	0.739	0.854	FALSE
Use Intention	CWAUI.UI01	0.899	0.754	0.837	0.902	0.839	FALSE
Use Intention	CWAUI.UI02	0.878	0.754	0.837	0.902	0.839	FALSE
Use Intention	CWAUI.UI03	0.826	0.754	0.837	0.902	0.839	FALSE

2.2.3 Discriminant validity

Discriminant validity is evaluated using the heterotrait-monotrait ratio (HTMT). The HTMT bootstrap confidence interval should not contain 1.

2.2.4 Results of the evaluation of the mode A measurement model

Convergent validity is problematic for Threat Appraisal. Specifically, the loading for the Perceived Vulnerability indicator EmpfVul.SQ003 is below the critical threshold of 0.4. Further, some other indicator loadings are below 0.708, but none below 0.4.

Internal consistency reliability is sufficient for all constructs except Perceived Threat.

Discriminant validity is problematic for the Perceived Goal Congruity and and Perceived Result Diagnosticity lower-order constructs. The upper bound of the HTMT confidence interval is above 1, implying some overlap.

2.2.5 Remedying the mode A measurement model problems

First, we test whether we can improve convergent validity for Threat Appraisal by dropping an indicator.

Deleting EmpfVul.SQ003 would raise alpha to 0.65. We will therefore delete the indicators EmpfVul.SQ003 to raise convergent validity and internal consistency reliability.

Table 2: Details of the bootstrapped HTMT ratios model iteration 1

Construct 1	Construct 2	Lower CI HTMT	Upper CI HTMT
Perceived Goal Congruity	Perceived Data Validity	0.615	0.835
Perceived Goal Congruity	Perceived Result Diagnosticity	0.858	1.013
Perceived Goal Congruity	Perceived Data Validity	0.615	0.835
Perceived Goal Congruity	Perceived Result Diagnosticity	0.858	1.013

Table 3: Cronbachs alpha for Perceived Vulnerability if an item is dropped

	raw_alpha	std.alpha
EmpfVul.SQ001	0.264	0.273
EmpfVul.SQ002	0.313	0.322
EmpfVul.SQ003	0.650	0.650

Second, we test whether we might improve discriminant validity for Perceived Goal Congruity and Perceived Result Diagnosticity by deleting indicators with a low correlation with other indicators of the same construct or with a high correlation with items measuring the other construct.

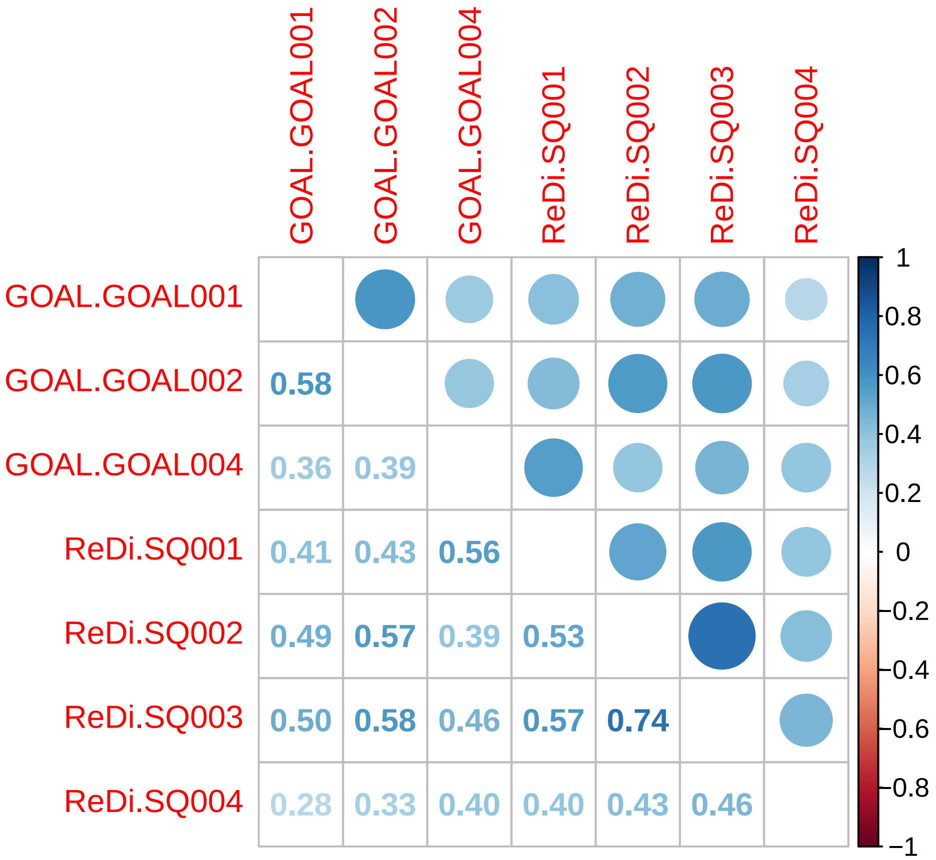

Indicators GOAL.GOAL001 and GOAL.GOAL002 are moderately correlated. Indicator GOAL.GOAL004 is more strongly correlated with Perceived Result Diagnosticity (PRD) indicators than with other Perceived Goal Congruity (PGC) indicators. With the exception of GOAL.GOAL004, ReDi.SQ001 is correlated more strongly with PRD than with PGC indicators. ReDi.SQ002 and ReDi.SQ003 are strongly correlated, but other than that similarly with PRD and PGC indicators. ReDi.SQ004 is not correlated strongly with anything, but overall more strongly with PRD indicators.

On that basis, one could start by removing GOAL.GOAL004 as an indicators and see whether that fixes the discriminant validity problems.

Table 4: Results of the mode B higher-order construct evaluation model iteration 1

HOC	LOC	VIF	Original.Est.Wt.	Bootstrap.Mean.Wt.	t(317) (weight)	0.in.Wt.CI	Original.Est.Ld.
Perceived Trustworthiness	Perceived Goal Congruity	2.127	0.240	0.243	3.499	FALSE	0.831
Perceived Trustworthiness	Perceived Data Validity	1.604	0.169	0.167	2.796	FALSE	0.711
Perceived Trustworthiness	Perceived Result Diagnosticity	2.318	0.701	0.697	10.789	FALSE	0.971

2.3 Evaluation of the mode B measurement model

There are no simple mode B constructs to evaluate.

2.4 Evaluation of the mode B higher-order constructs

2.4.1 Convergent validity

Convergent validity cannot be evaluated.

2.4.2 Collinearity

The variance inflation factor (VIF) should be < 5, ideally ≤ 3.

2.4.3 Significance and relevance

Indicator weights should be significant ($t \geq 1.65$, bootstrapping CI does not include 0). Otherwise, the loading l should be ≥ 0.50. Any positive indicator weight implies relevance.

2.4.4 Results of the mode B higher-order constructs evaluation

For the mode B higher-order constructs, there are no issues with collinearity (VIF below 3). All weights are significant and relevant. The mode B higher-order constructs can be retained as hypothesized.

2.5 Results summary

To improve convergent validity and internal consistency reliability, EmpfVul.SQ003 and potentially GOAL.GOAL004 should be deleted. Other than that, the mode A measurement model can be retained as-is. The mode B higher-order construct can also be retained as-is.

3 Iteration 2 Measurement Model Evaluation

3.1 Model plots

3.1.1 Original Estimate Path Model

This is the original estimate path model.

3.1.2 Bootstrapped Path Model

This is the bootstrapped path model.

3.1.3 Measurement Model Only

This is a path model showing only the measurement model components.

3.2 Evaluation of the mode A measurement model

3.2.1 Convergent validity

Ideally, outer loadings (l) should be ≥ 0.70. Loadings below 0.40 are unacceptable. AVE should be > 0.50.

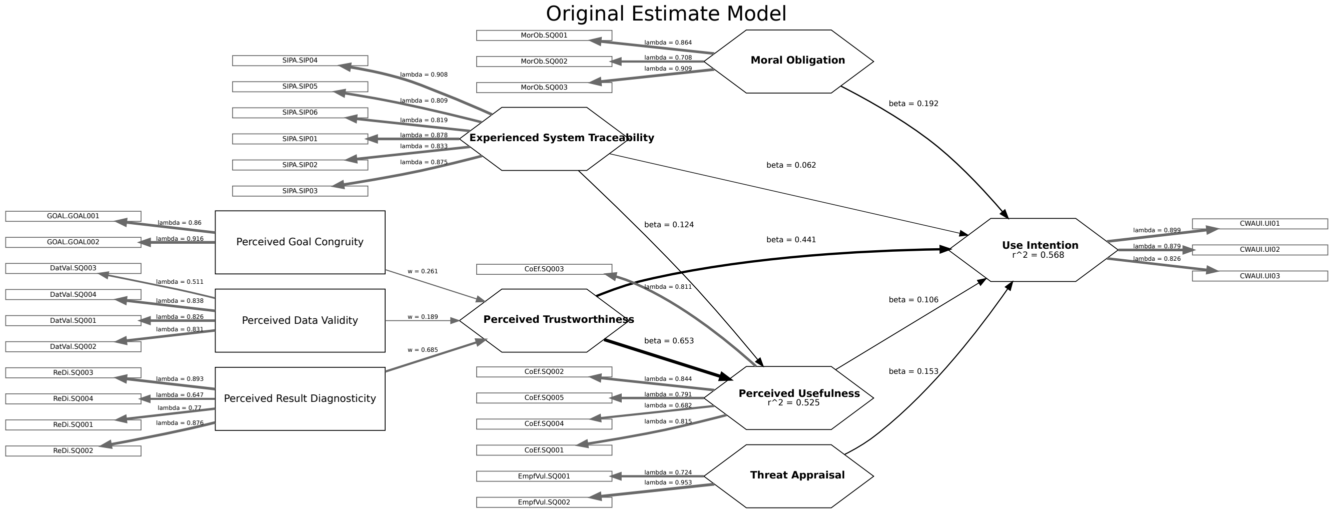

Figure 4: Original estimate model iteration 2

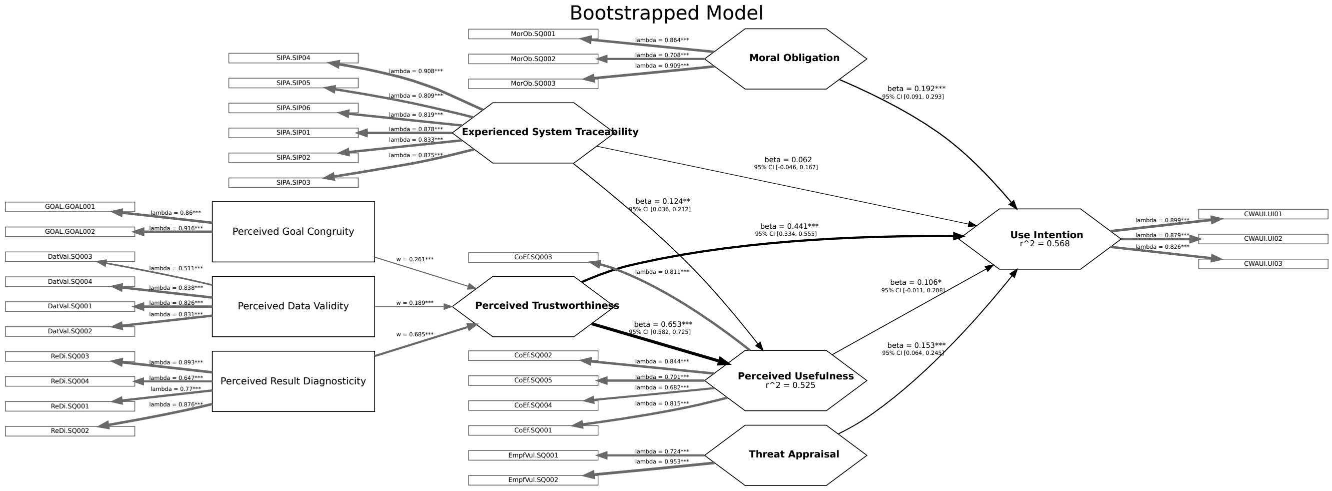

Figure 5: Bootstrapped model iteration 2

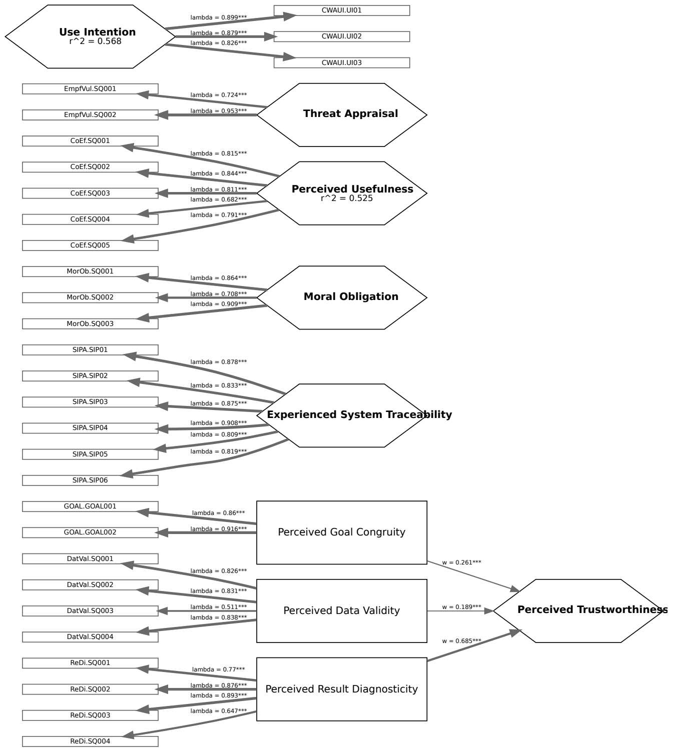

Figure 6: Only measurement model iteration 2

3.2.2 Internal consistency reliability

Both Cronbach's a and composite reliability rc should be ≥ 0.60 and ≤ 0.90. The upper threshold of acceptability is 0.95.

3.2.3 Discriminant validity

Discriminant validity is evaluated using the heterotrait-monotrait ratio (HTMT). The HTMT bootstrap confidence interval should not contain 1.

3.2.4 Results of the evaluation of the mode A measurement model

Convergent validity is sufficient for all constructs. Some indicator loadings are below 0.708, but none below 0.4.

Internal consistency reliability is also sufficient for all constructs, as is discriminant validity.. The upper bound of the HTMT confidence interval for the Perceived Goal Congruity and and Perceived Result Diagnosticity lower-order constructs is still at 0.90 but because the constructs are strongly related, we judge this to be acceptable.

3.3 Evaluation of the mode B measurement model

There are no simple mode B constructs to evaluate.

3.4 Evaluation of the mode B higher-order constructs

3.4.1 Convergent validity

Convergent validity cannot be evaluated.

3.4.2 Collinearity

The variance inflation factor (VIF) should be < 5, ideally ≤ 3.

Table 5: Results of the mode A measurement model evaluation model iteration 2

Construct	Indicator	Loading	AVE	Calpha	rhoC	rhoA	1.in.HTMT.CI
Perceived Goal Congruity	GOAL.GOAL001	0.860	0.789	0.736	0.882	0.765	FALSE
Perceived Goal Congruity	GOAL.GOAL002	0.916	0.789	0.736	0.882	0.765	FALSE
Perceived Data Validity	DatVal.SQ001	0.826	0.584	0.759	0.844	0.818	FALSE
Perceived Data Validity	DatVal.SQ002	0.831	0.584	0.759	0.844	0.818	FALSE
Perceived Data Validity	DatVal.SQ003	0.511	0.584	0.759	0.844	0.818	FALSE
Perceived Data Validity	DatVal.SQ004	0.838	0.584	0.759	0.844	0.818	FALSE
Perceived Result Diagnosticity	ReDi.SQ001	0.770	0.644	0.813	0.877	0.852	FALSE
Perceived Result Diagnosticity	ReDi.SQ002	0.876	0.644	0.813	0.877	0.852	FALSE
Perceived Result Diagnosticity	ReDi.SQ003	0.893	0.644	0.813	0.877	0.852	FALSE
Perceived Result Diagnosticity	ReDi.SQ004	0.647	0.644	0.813	0.877	0.852	FALSE
Experienced System Traceability	SIPA.SIP01	0.878	0.730	0.926	0.942	0.928	FALSE
Experienced System Traceability	SIPA.SIP02	0.833	0.730	0.926	0.942	0.928	FALSE
Experienced System Traceability	SIPA.SIP03	0.875	0.730	0.926	0.942	0.928	FALSE
Experienced System Traceability	SIPA.SIP04	0.908	0.730	0.926	0.942	0.928	FALSE
Experienced System Traceability	SIPA.SIP05	0.809	0.730	0.926	0.942	0.928	FALSE
Experienced System Traceability	SIPA.SIP06	0.819	0.730	0.926	0.942	0.928	FALSE
Perceived Usefulness	CoEf.SQ001	0.815	0.625	0.849	0.892	0.855	FALSE
Perceived Usefulness	CoEf.SQ002	0.844	0.625	0.849	0.892	0.855	FALSE
Perceived Usefulness	CoEf.SQ003	0.811	0.625	0.849	0.892	0.855	FALSE
Perceived Usefulness	CoEf.SQ004	0.683	0.625	0.849	0.892	0.855	FALSE
Perceived Usefulness	CoEf.SQ005	0.791	0.625	0.849	0.892	0.855	FALSE
Moral Obligation	MorOb.SQ001	0.864	0.692	0.776	0.870	0.825	FALSE
Moral Obligation	MorOb.SQ002	0.708	0.692	0.776	0.870	0.825	FALSE
Moral Obligation	MorOb.SQ003	0.909	0.692	0.776	0.870	0.825	FALSE
Threat Appraisal	EmpfVul.SQ001	0.724	0.716	0.650	0.832	0.966	FALSE
Threat Appraisal	EmpfVul.SQ002	0.953	0.716	0.650	0.832	0.966	FALSE
Use Intention	CWAUI.UI01	0.899	0.754	0.837	0.902	0.839	FALSE
Use Intention	CWAUI.UI02	0.879	0.754	0.837	0.902	0.839	FALSE
Use Intention	CWAUI.UI03	0.826	0.754	0.837	0.902	0.839	FALSE

Table 6: Results of the mode B higher-order construct evaluation model iteration 2

HOC	LOC	VIF	Original.Est.Wt.	Bootstrap.Mean.Wt.	t(317) (weight)	0.in.Wt.CI	Original.Est.Ld.
Perceived Trustworthiness	Perceived Goal Congruity	1.739	0.261	0.262	4.326	FALSE	0.787
Perceived Trustworthiness	Perceived Data Validity	1.547	0.189	0.188	3.229	FALSE	0.707
Perceived Trustworthiness	Perceived Result Diagnosticity	2.157	0.685	0.683	11.383	FALSE	0.965

3.4.3 Significance and relevance

Indicator weights should be significant (t ≥ 1.65, bootstrapping CI does not include 0). Otherwise, the loading l should be ≥ 0.50. Any positive indicator weight implies relevance.

3.4.4 Results of the mode B higher-order constructs evaluation

For the mode B higher-order constructs, there are no issues with collinearity (VIF below 3). All weights are significant and relevant. The mode B higher-order constructs can be retained as hypothesized.

3.5 Results summary

The measurement model can be retained as-is. Structural model evaluation can commence.

4 Iteration 2 Structural Model Evaluation

4.1 Model plots

4.1.1 Structural Model Only

This is a path model showing only the structural model components.

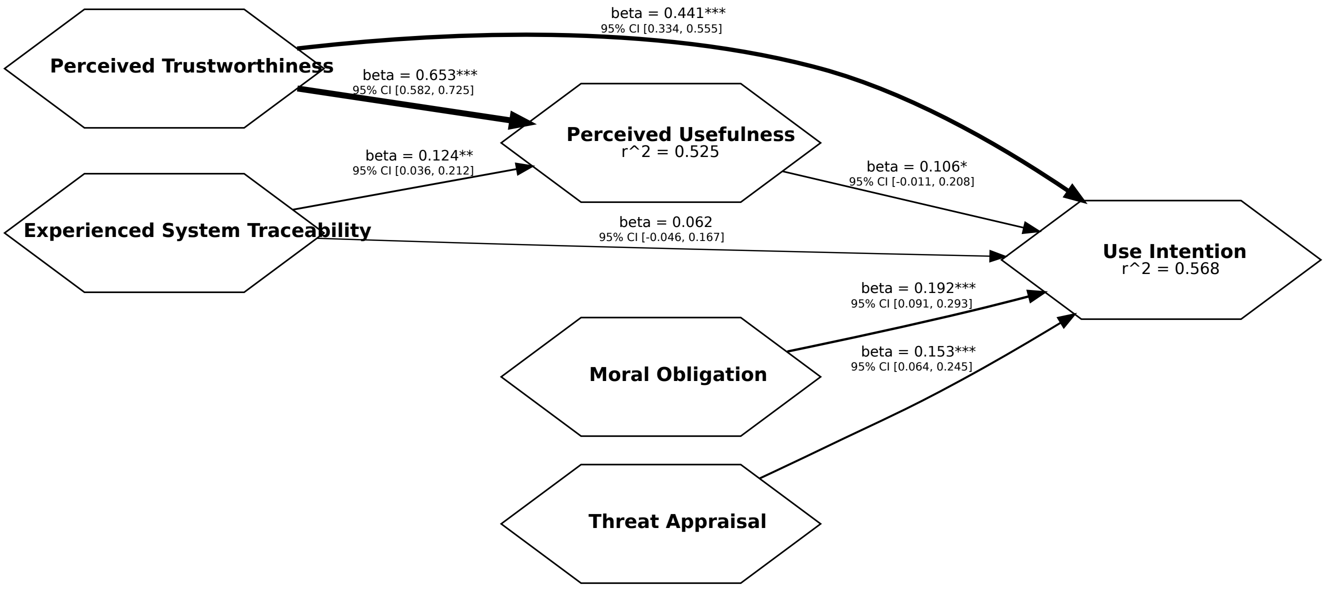

Figure 7: Only structural model iteration 2

4.2 Collinearity

Collinearity is assessed using the variance inflation factor (VIF). VIF should be < 5, ideally ≤ 3.

VIF is not above 3 for any relationship, implying no critical collinearity.

Table 7: Results of the structural collinearity assessment model iteration 2

Exogenous.Construct	Endogenous.Construct	VIF
Perceived Trustworthiness	Perceived Usefulness	1.355
Experienced System Traceability	Perceived Usefulness	1.355
Perceived Trustworthiness	Use Intention	2.505
Experienced System Traceability	Use Intention	1.409
Moral Obligation	Use Intention	1.708
Perceived Usefulness	Use Intention	2.163
Threat Appraisal	Use Intention	1.221

Table 8: Total indirect effects model iteration 2

Exogenous	Mediator	Endogenous	Original Est.	Bootstrap Mean	Bootstrap SD	T Stat.	2.5% CI	97.5% CI
Perceived Trustworthiness	Perceived Usefulness	Use Intention	0.069	0.066	0.037	1.867	-0.007	0.139
Experienced System Traceability	Perceived Usefulness	Use Intention	0.013	0.013	0.009	1.457	-0.001	0.034

4.3 Mediation analysis

For the mediation analysis, we first analyze all total indirect effects.

Both total indirect effect confidence intervals contain 0, implying a lack of significance. This points to a lack of mediation. Further below, we will test whether there is a direct effect of the exogenous constructs on Use Intention that is not mediated by Perceived Usefulness.

4.4 Significance and relevance of path coefficients

Significance is denoted by t-test and p values. A t of ≥ 1.65 signifies significance at the 10 % level, t ≥ 1.96 at the 5 % level and t ≥ 2.57 at the 1 % level.

The findings on the significance and relevance of paths mirror the findings on the effects. The path coefficients for the path from Experienced System Traceability to Use Intention is very small and non-significant on basis of both the t and p-value and 0 being in the bootstrapping confidence interval. The path coefficient from Perceived Usefulness to Use Intention is also non-significant on the basis of 0 being in the bootstrapping confidence interval, but the t and p-value imply significance.

4.5 In-sample predictive power

In-sample predictive power is assessed using variance explained R^2. $R^2 \geq 0.75$ indicates substantial in-sample predictive power, $R^2 \geq 0.5$ moderate and $R^2 \geq 0.25$ weak in-sample predictive power. $R^2 \leq 0.10$ indicates a lack of model predictiveness.

All R^2 values are above 0.5, indicating moderate in-sample predictive power.

4.6 Effect size

Effect size $f2^2$ measures the impact of a predictor construct on an endogenous construct. $f^2 \geq 0.35$ indicates a large effect, $f^2 \geq 0.15$ a medium and $f^2 \geq 0.02$ a small effect.

Table 9: Relevance and significance of path coefficients model iteration 2

Exogenous Construct	Endogenous Construct	Original Est.	Bootstrap Mean	Bootstrap SD	2.5% CI	97.5% CI	0 in CI	t(317)	p
Perceived Trustworthiness	Perceived Usefulness	0.653	0.656	0.037	0.582	0.725	FALSE	17.471	0.000
Perceived Trustworthiness	Use Intention	0.441	0.446	0.056	0.334	0.555	FALSE	7.869	0.000
Experienced System Traceability	Perceived Usefulness	0.124	0.124	0.045	0.036	0.212	FALSE	2.768	0.003
Experienced System Traceability	Use Intention	0.062	0.061	0.054	-0.046	0.167	TRUE	1.144	0.127
Moral Obligation	Use Intention	0.192	0.192	0.051	0.091	0.293	FALSE	3.740	0.000
Perceived Usefulness	Use Intention	0.106	0.101	0.056	-0.011	0.208	TRUE	1.901	0.029
Threat Appraisal	Use Intention	0.153	0.155	0.047	0.064	0.245	FALSE	3.286	0.001

Table 10: In-sample predictive power model iteration 2

Construct	R.2	AdjR.2
Perceived Usefulness	0.525	0.522
Use Intention	0.568	0.561

Table 11: Effect sizes model iteration 2

Exogenous Construct	Endogenous Construct	f^2
Perceived Trustworthiness	Perceived Usefulness	0.658
Experienced System Traceability	Perceived Usefulness	0.024
Perceived Trustworthiness	Use Intention	0.179
Experienced System Traceability	Use Intention	0.006
Moral Obligation	Use Intention	0.050
Perceived Usefulness	Use Intention	0.012
Threat Appraisal	Use Intention	0.044

4.7 Summary table

4.8 Results summary

The coefficients of the paths from Experienced System Traceability and Perceived Usefulness to Use Intention are very small and non-significant. Also, the effect sizes are negligible. Therefore, these paths should be removed from the model. Similarly, there is a negligible effect size for the path from Experienced System Traceability to Perceived Usefulness, but the path coefficient is significant. For now, the path will remain in the model.

5 Iteration 3 Structural Model Evaluation

5.1 Model plots

5.1.1 Structural Model Only

This is a path model showing only the structural model components.

5.2 Collinearity

Collinearity is assessed using the variance inflation factor (VIF). VIF should be < 5, ideally ≤ 3.

VIF is not above 3 for any relationship, implying no critical collinearity.

5.3 Significance and relevance of path coefficients

Significance is denoted by t-test and p values. A t of ≥ 1.65 signifies significance at the 10 % level, t ≥ 1.96 at the 5 % level and t ≥ 2.57 at the 1 % level.

All path coefficients are significant at $p < .05$ and above 0.1.

Table 12: Structural model summary model iteration 2

Exogenous Construct	Endogenous Construct	Original Est.	Bootstrap Mean	0 in CI	Path Coefficient t(317)	p	f^2	VIF
Perceived Trustworthiness	Perceived Usefulness	0.653	0.656	FALSE	17.471	0.000	0.658	1.355
Perceived Trustworthiness	Use Intention	0.441	0.446	FALSE	7.869	0.000	0.179	2.505
Experienced System Traceability	Perceived Usefulness	0.124	0.124	FALSE	2.768	0.003	0.024	1.355
Experienced System Traceability	Use Intention	0.062	0.061	TRUE	1.144	0.127	0.006	1.409
Moral Obligation	Use Intention	0.192	0.192	FALSE	3.740	0.000	0.050	1.708
Perceived Usefulness	Use Intention	0.106	0.101	TRUE	1.901	0.029	0.012	2.163
Threat Appraisal	Use Intention	0.153	0.155	FALSE	3.286	0.001	0.044	1.221

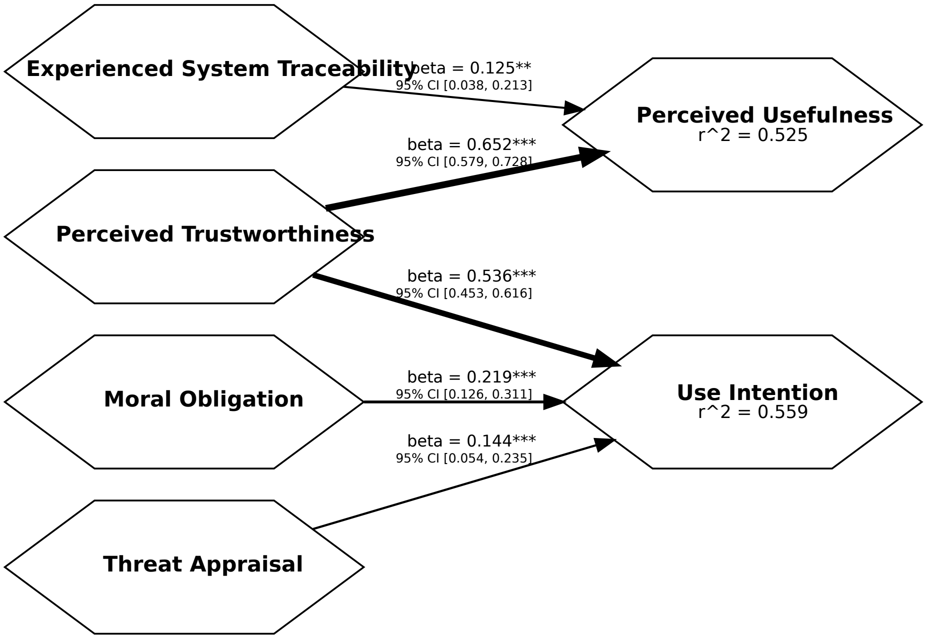

Figure 8: Only structural model iteration 3

Table 13: Results of the structural collinearity assessment model iteration 3

Exogenous.Construct	Endogenous.Construct	VIF
Perceived Trustworthiness	Perceived Usefulness	1.356
Experienced System Traceability	Perceived Usefulness	1.356
Perceived Trustworthiness	Use Intention	1.471
Moral Obligation	Use Intention	1.628
Threat Appraisal	Use Intention	1.212

Table 14: Relevance and significance of path coefficients model iteration 3

Exogenous Construct	Endogenous Construct	Original Est.	Bootstrap Mean	Bootstrap SD	2.5% CI	97.5% CI	0 in CI	t(317)	p
Perceived Trustworthiness	Perceived Usefulness	0.652	0.656	0.038	0.579	0.728	FALSE	17.242	0.000
Perceived Trustworthiness	Use Intention	0.536	0.539	0.041	0.453	0.616	FALSE	12.959	0.000
Experienced System Traceability	Perceived Usefulness	0.125	0.125	0.045	0.038	0.213	FALSE	2.795	0.003
Moral Obligation	Use Intention	0.219	0.218	0.048	0.126	0.311	FALSE	4.602	0.000
Threat Appraisal	Use Intention	0.144	0.145	0.046	0.054	0.235	FALSE	3.125	0.001

Table 15: In-sample predictive power model iteration 3

Construct	R.2	AdjR.2
Perceived Usefulness	0.525	0.522
Use Intention	0.559	0.555

Table 16: Effect sizes model iteration 3

Exogenous Construct	Endogenous Construct	f^2
Perceived Trustworthiness	Perceived Usefulness	0.648
Experienced System Traceability	Perceived Usefulness	0.024
Perceived Trustworthiness	Use Intention	0.439
Moral Obligation	Use Intention	0.067
Threat Appraisal	Use Intention	0.039

5.4 In-sample predictive power

In-sample predictive power is assessed using variance explained R^2. $R^2 \geq 0.75$ indicates substantial in-sample predictive power, $R^2 \geq 0.5$ moderate and $R^2 \geq 0.25$ weak in-sample predictive power. $R^2 \leq 0.10$ indicates a lack of model predictiveness.

All R^2 values are above 0.5, indicating moderate in-sample predictive power.

5.5 Effect size

Effect size $f2^2$ measures the impact of a predictor construct on an endogenous construct. $f^2 \geq 0.35$ indicates a large effect, $f^2 \geq 0.15$ a medium and $f^2 \geq 0.02$ a small effect.

The effects for the paths from Experienced System Traceability to Perceived Usefulness, and from Threat Appraisal and Moral Obligation to Use Intention are small. The effects for the paths from Perceived Trustworthiness to both Use Intention and Perceived Usefulness are large.

5.6 Summary table

5.7 Results summary

All paths are significant and relevant and effects are small at a minimum. The model can be retained as is.

5.8 Achieved power

To calculate the achieved power of our model, we use the inverse square root method described by Kock and Hadaya (2018). [1] "The achieved power given the achieved sample size of 317, a 5% significance level, and the smallest effect size in this model being 0.125 is 0.72."

Table 17: Structural model summary model iteration 3

Exogenous Construct	Endogenous Construct	Original Est.	Bootstrap Mean	0 in CI	Path Coefficient t(317)	p	f^2	VIF
Perceived Trustworthiness	Perceived Usefulness	0.652	0.656	FALSE	17.242	0.000	0.648	1.356
Perceived Trustworthiness	Use Intention	0.536	0.539	FALSE	12.959	0.000	0.439	1.471
Experienced System Traceability	Perceived Usefulness	0.125	0.125	FALSE	2.795	0.003	0.024	1.356
Moral Obligation	Use Intention	0.219	0.218	FALSE	4.602	0.000	0.067	1.628
Threat Appraisal	Use Intention	0.144	0.145	FALSE	3.125	0.001	0.039	1.212
