# Supplementary material for: Effects of User Experience in Automated Information Processing on Perceived Usefulness of Digital Contact-Tracing Apps: Cross-Sectional Survey Study
Source: JMIR Hum Factors. 2024 Jun 25;11:e53940. doi: 10.2196/53940 (PMC11234054; doi:10.2196/53940)
Supplement: Multimedia Appendix 3 [file humanfactors_v11i1e53940_app3.pdf]

Appendix - Detailed Coding Scheme

Top Code	Sub Code	Definition	Anchoring example (original from German)
**Contact/ Risk Information**	/		
	**Time-related Information**		
	Time period of contact	The time period when a registered contact happened, can also be an exact point in time	Wann (…) bin ich einer Person begegnet? (1, Pos. 1)
	Duration of contact	The duration of a registered contact	Wie lange hatte ich Kontakt mit der Person in derselben Umgebung? (1, Pos. 1)
	Time since contact	The time passed since a contact happened	Wann fand der Kontakt statt?
	Time period of possible contact tracing	Period during which contact tracing was possible at all (e.g. cell phone present)	wann habe ich das Handy wirklich bei mir
	**Location-related information**		
	Location of Contact	The (exact GPS) location of the contact	(…) wo hatte ich (…) Kontakt zu einer Risiko-Person? (44, Pos. 1)
	Direct/Indirect	Whether the contact was direct (i.e. without walls, glass or other blocking materials) or indirect (i.e. no direct line between faces possible)	War es eine direkte Begegnung oder saßen wir in unterschiedlichen Autos? (273, Pos. 1)
	Outdoor/Indoor	Whether the place of contact was indoor or outdoor	War ich in einem geschlossenen Raum? (40, Pos. 1)
	**Exposition-related Information**		
	Mask Status	Whether the user wore a mask or not	Maskenpflicht? (4, Pos. 1)
	Distance to Warning Person	Distance between me and the contact	genauer Abstand (14, Pos. 1)

Top Code	Sub Code	Definition	Anchoring example (original from German)
	Amount of Devices/People when exposed	Number of devices present during contact	mit wie vielen Geräten bestand der Kontakt (131, Pos. 1)
	Hygiene precautions	Hygienic precautions, implemented on site	Konkreten Ort mitteilen (geschlossener Raum, frische luft) oder hygienemaßnahmen der Umgebung.
	Unspecified Scenarios	Other Information assisting the user in asserting his/her risk, topics may overlap with different codes of exposition-related information but are not sufficiently specific	Welche Begegnung hat dazu geführt? (ggf. problematisch für die Anonymität, dennoch wäre z.B. ein Zeitraum <24h hilfreich, um den Ort der Begegnung einschätzen und/oder Menschen ohne App/Warnung darauf hinweisen zu könnnen) (97, Pos. 1)
	**Action-related Information**	Possible and suggested course of action	Was muss ich jetzt tun? (195, Pos. 1)
	**Information related to warning person**		
	Time of Test	Time when the infected person tested positive	Wann wurde das positive Testergebnis der Person eingetragen? (243, Pos. 1)
	Duration of infection	Point in time when the person through which the warning occurred became infected	Seit wann ist die positiv getestete Person infiziert? (146, Pos. 1)
	Symptoms in the person	The first time the person for whom my contact led to a warning experienced symptoms	Wann traten bei dem Kontakt die ersten Symptome auf? (177, Pos. 5)
	Vaccination status	Vaccination status of the person	Impfstatus sollte in Risiko-Berechnung berücksichtigt werden
	Corona Variant	Corona variant (e.g. Omikron) with which the infected person is infected.	virusvariante (258, Pos. 1)
**Pandemic Related Information**	**Statistics**		
	Number of Deaths	The number of COVID-19 deaths	Anzahl der Todesfälle, anstatt nur die Erkrankungen

Top Code	Sub Code	Definition	Anchoring example (original from German)
			anzugeben. (260, Pos. 2)
	Number of Infections	The number of new COVID-19 infections	Kein Beispiel?
**App Related Information**	**Number of Users**	The number of users of the CWA	Ist das Risiko jetzt wirklich so niedrig oder nutzen die App einfach weniger Leute als noch letztes Jahr? (37, Pos. 1)
	**General Calculation-related information**		
	Reasons for Status Change	Reasons for the change in risk calculation	Wie genau die Risikoermittlung funktioniert, d.h. wie müssen Abstand und Zeit zu einer positiv getesteten Person tatsächlich ausfallen, damit ich eine Meldung erhalte und der Status geändert wird. (39, Pos. 1)
	Calculation parameters	Parameters used by the system for the calculations	Berechnungsgrundlage für erhöhtes Risiko ist sehr schwer herauszufinden (233, Pos. 1)
	**Certainty about result**	Certainty of the results calculated by the system	Wie sicher ist das Ergebnis? (193, Pos. 1)
	**Integration of Tests (self and externally administered)**	The ability to enter or delete results from tests in the app	Dass ich Corona-positiv bin, ohne einen PCR Test gemacht zu haben.
	**Linking with private data**	Possibility to link the app functionalities with private data	Lokal Kontakte aus Telefonbuch zu Tagebucheinträgen hinzufügen
**Others**		Everything that does not fit any category	Bsp: Ich wurde nach einem Messebesuch gewarnt mit roter Anzeige. Es macht für mich einen Unterschied, ob die Warnung aus einer Situation bei der Zugfahrt (ich hatte eine FFP2 Maske an, keine Gespräche mit den anderen Fahrgästen) oder bei der Messe (längeres Gespräch ohne Maske mit einer Person) entstanden ist. In der ersten Situation hätte

Top Code	Sub Code	Definition	Anchoring example (original from German)
			ich mich kaum gefährdet gefühlt, in der zweiten Situation in sehr hohem Maße (8, Pos. 3)
**Assumed reasons for perceived information processing**	/	Reasons for missing feature integration, e.g. data protection	Aber vermutlich datenschutztechnisch nicht umsetzbar, weil der Kontakt sonst ggf. durch mich identifizierbar wäre?

Appendix - Descriptive Data

Information demand	Automation2Human		Human2Automation	
	n	%	*n*	%
Contact/Risk	196	93	62	82
Time	111	57	8	13
Location	45	23	24	39
Exposition	30	15	22	35
Action	3	2	0	0
Warning person	7	4	8	13
App	14	7	14	18
Private data linking	0	0	2	14
User number	1	7	0	0
General calculation	12	86	0	0
Result certainty	1	7	0	0
Test integration	0	0	12	86
Pandemic	1	1	0	0
Statistics	1	100	0	0

Note. $N = 287$ ($n = 211$ for Machine2Human and $n = 76$ for Human2Machine).
